# P2RY13-Negative Tumor-Associated Macrophages Promote NETosis and Are Associated with Poor Prognosis Across Multiple Cancers

**DOI:** 10.3390/cancers18152500

**Published:** 2026-08-04

**Authors:** Shen Yang, Guangsheng Zhu, Zixuan Hu, Mingbiao Li, Jianfang Wang, Zhanrui Zhang, Jun Chen, Renwang Liu

**Affiliations:** 1 Department of Lung Cancer Surgery, Center of Thoracic Surgery, Tianjin Medical University General Hospital, Tianjin 300052, China; 2 Tianjin Key Laboratory of Lung Cancer Metastasis and Tumor Microenvironment, Tianjin Lung Cancer Institute, Tianjin Medical University General Hospital, Tianjin 300052, China; 3 Department of Esophagus Surgery, Key Laboratory of Cancer Prevention and Therapy, National Clinical Research Center of Cancer, Tianjin Medical University Cancer Institute and Hospital, Tianjin 300060, China

**Keywords:** P2RY13, tumor-associated macrophages, neutrophil extracellular traps, NETosis, Pan-cancer analysis

## Abstract

**Background**: Neutrophil extracellular traps (NETs) promote tumor progression and immune evasion across multiple cancers. However, the mechanisms governing NET formation (NETosis) within the tumor immune microenvironment remain incompletely understood. Low P2RY13 expression is associated with the pro-tumor activity of neutrophils in lung adenocarcinoma. **Methods**: Pan-cancer bioinformatics analyses were performed, including differential gene expression, prognostic, tumor-infiltrating immune cell, and NET scoring analyses. Single-cell profiling was conducted using the Tumor Immune Single-cell Hub 2 database. Immunohistochemical (IHC) and multiplex immunofluorescence (mIF) staining of tissue microarrays (TMAs) was performed to validate findings in lung adenocarcinoma (LUAD), liver hepatocellular carcinoma (LIHC), and colorectal cancer (CRC). In vitro validation was conducted using gene modulation, tumor-cell-conditioned medium (CM) education, tumor-associated macrophage (TAM)-like macrophage CM transfer, immunofluorescence staining, and ELISA. **Results:** Bioinformatics analyses suggested that P2RY13 is frequently dysregulated in multiple cancers and that low P2RY13 expression is associated with poor prognosis and reduced immunotherapy responsiveness. Single-cell analyses revealed that P2RY13 is predominantly expressed in TAMs rather than tumor cells. Macrophages without detectable P2RY13 transcripts were markedly enriched in tumor tissues compared with paired normal tissues, and their high infiltration appeared to be associated with elevated NET scores. Tissue microarray-based IHC and mIF analyses further validated this infiltration pattern in LUAD, LIHC, and CRC. In vitro, CM from P2RY13-silenced, tumor-educated TAM-like macrophages enhanced neutrophil NETosis, whereas P2RY13 re-expression attenuated this effect. Furthermore, TMA staining indicated a positive correlation between P2RY13^−^CD68^+^ TAM infiltration and NET expression in the three malignancies. **Conclusions**: We identified a potentially conserved NETosis-regulating pattern in multiple cancers in which P2RY13-negative TAMs promote NET formation and are associated with adverse clinical outcomes. These findings suggest a previously unrecognized tumor-promoting mechanism and highlight potential therapeutic targets for cancer treatment.

## Simple Summary

Neutrophil extracellular traps (NETs) promote tumor progression, yet the mechanisms underlying NET formation (NETosis) within the tumor immune microenvironment remain incompletely understood. Low P2RY13 expression has been linked to pro-tumor neutrophils in lung adenocarcinoma. In this study, pan-cancer bioinformatics analyses showed that reduced P2RY13 expression is commonly associated with poor prognosis. Single-cell and tissue microarray analyses further suggested that P2RY13-negative tumor-associated macrophages (TAMs) are highly enriched in tumor tissues in multiple cancers, including lung adenocarcinoma, liver hepatocellular carcinoma, and colorectal cancer. Increased infiltration of these TAMs was associated with enhanced NET formation. In vitro experiments further confirmed that P2RY13-silenced TAM-like macrophages induce neutrophil NETosis. These findings suggest a potential mechanism by which P2RY13-negative TAMs promote NET formation and contribute to adverse clinical outcomes, providing potential therapeutic insights.

## 1. Introduction

Neutrophils are increasingly recognized as active regulators of tumor progression rather than passive bystanders within the tumor immune microenvironment (TIME) [1,2]. Beyond their conventional roles in antimicrobial defense and acute inflammation, neutrophils can acquire tumor-promoting properties through the release of neutrophil extracellular traps (NETs), a process referred to as NET formation or NETosis [3,4]. NETs are web-like extracellular structures composed of decondensed chromatin decorated with granular and cytoplasmic proteins, including myeloperoxidase (MPO), neutrophil elastase, and citrullinated histone H3 (citH3) [5]. Accumulating evidence indicates that NETs facilitate tumor cell invasion, metastasis, vascular adhesion, thrombosis, and immune evasion in multiple cancer types [6,7]. Mechanistically, NETs may promote tumor progression by trapping circulating tumor cells, remodeling the extracellular matrix, amplifying inflammatory signaling, and impairing antitumor immune responses [8,9,10]. Despite these advances, the upstream cellular and molecular mechanisms sustaining NETosis within the TIME remain incompletely understood.

Neutrophils within the tumor microenvironment do not function in isolation [11]. Their recruitment, activation, and phenotypic remodeling depend on dynamic interactions with tumor cells, tumor-associated macrophages (TAMs), stromal cells, and other immune populations [12,13,14]. In particular, TAMs serve as major regulators of local inflammatory networks within the TIME. Through soluble mediators and cell–cell communication, TAMs can influence neutrophil polarization toward N1 or N2 phenotypes, thereby modulating their biological behavior [15]. Previous studies have shown that TAMs can induce NETosis; however, the specific TAM subtypes responsible for this process, along with their defining molecular markers and functional characteristics, remain poorly characterized.

Our previous work demonstrated that P2RY13 is significantly downregulated in lung adenocarcinoma (LUAD) and associated with poor prognosis [16]. Its expression has also been linked to the pro-tumor activity of neutrophils, suggesting a potential role in regulating myeloid cell interactions within the tumor microenvironment. P2RY13 is a G protein-coupled purinergic receptor responsive to extracellular adenosine diphosphate (ADP) signaling that has been implicated in immune regulation, inflammatory responses, and cellular metabolic homeostasis [17,18]. Purinergic signaling constitutes an important component of the inflammatory microenvironment, in which extracellular nucleotides function as danger-associated molecular signals that regulate immune cell activation, migration, and effector functions [19,20]. Accordingly, P2RY13 may act not only as a prognosis-associated molecule in cancer but also as an immune regulatory factor within the tumor microenvironment.

Emerging evidence further supports a role for P2RY13 in tumor immune regulation. For example, Lan et al. reported that P2RY13-positive dendritic cells exhibit enhanced antigen-presenting capacity and stronger interactions with lymphocytes, particularly T cells, suggesting a potential role for P2RY13 in promoting antitumor immune responses [21]. However, the expression pattern and functional significance of P2RY13 in TAMs remain insufficiently characterized. In particular, whether P2RY13 regulates TAM–neutrophil interactions and contributes to NETosis remains unclear.

Accordingly, the present study was conducted to better understand the mechanisms governing NETosis within the TIME. By using single-cell transcriptomic analysis, we identified minimal P2RY13 expression in tumor cells and predominant enrichment within TAM populations. Moreover, by integrating bioinformatic analyses, in vitro functional validation, and tissue-level validation using tissue microarrays from three common cancer types, a broad pro-NETosis role of P2RY13-negative TAMs in multiple tumors was preliminarily identified and validated. These findings provide new insights into the TAM-mediated regulation of NETosis and highlight potential therapeutic targets for pan-cancer treatment.

## 2. Materials and Methods

### 2.1. Pan-Cancer Transcriptomic Data Acquisition and Processing

Pan-cancer transcriptomic and clinical data were obtained from the SangerBox platform (http://sangerbox.com/login.html, accessed on 13 April 2026) [22], which integrates uniformly processed datasets from The Cancer Genome Atlas (TCGA) and the Genotype-Tissue Expression (GTEx) project. TCGA tumor RNA-sequencing data and TCGA/GTEx normal tissue data were used to compare P2RY13 expression between tumor and normal tissues among cancer types. For paired analyses, only TCGA samples with matched tumor and adjacent normal tissues were included.

Expression values were log2-transformed according to the SangerBox data-processing pipeline when appropriate. Cancer type abbreviations followed TCGA nomenclature. LUAD, liver hepatocellular carcinoma (LIHC), and colorectal cancer (CRC)-related cohorts, represented by the combined TCGA COADREAD cohort, were selected for subsequent analyses because P2RY13 exhibited consistent downregulation in these tumor types.

### 2.2. Differential Expression Analysis of P2RY13

Differential expression analysis of P2RY13 between tumor and normal tissues was performed using SangerBox (http://sangerbox.com/login.html, accessed on 13 April 2026). For pan-cancer unpaired analyses, TCGA tumor samples were compared with corresponding normal tissues from TCGA and/or GTEx. For paired analyses, matched tumor and adjacent normal samples from TCGA were evaluated. Statistical significance was assessed using the default methods implemented in SangerBox, and results were visualized as box plots or paired comparison plots.

### 2.3. Survival Analysis

The prognostic significance of P2RY13 expression was evaluated using TCGA clinical data through SangerBox (http://sangerbox.com/login.html, accessed on 13 April 2026). Survival endpoints included overall survival (OS), progression-free interval (PFI), disease-specific survival, and disease-free interval. Pan-cancer Cox proportional hazards regression analysis was performed to estimate hazard ratios (HRs) and 95% confidence intervals (CIs). An HR < 1 indicated that higher P2RY13 expression was associated with a lower risk of death or disease progression.

For Kaplan–Meier survival analyses, patients were stratified into high- and low-expression groups according to the P2RY13 expression cutoff implemented in SangerBox. Survival differences between groups were assessed using the log-rank test. Representative Kaplan–Meier survival curves were generated for LUAD, LIHC, and COADREAD.

### 2.4. Immune Microenvironment and Immune Infiltration Analysis

Precomputed immune microenvironment-related data were downloaded from the SangerBox platform (http://sangerbox.com/login.html, accessed on 13 April 2026), including ESTIMATE-derived Stromal Score, Immune Score, and ESTIMATE Score, as well as immune cell infiltration profiles generated using quanTIseq, MCPcounter, and EPIC based on TCGA transcriptomic data. Following data export, correlations between P2RY13 expression and immune scores or immune cell infiltration levels were analyzed locally using R (version 3.6.4). Correlation coefficients and *p* values were calculated among cancer types, and results were visualized as heatmaps.

### 2.5. Analysis of Immunomodulatory Molecules and Immunophenoscore

Immunomodulatory gene expression data and immunophenoscore-related metrics were downloaded from the SangerBox platform (http://sangerbox.com/login.html, accessed on 13 April 2026). Correlations between P2RY13 expression and immune regulatory genes or immunophenoscore metrics were analyzed using R (version 3.6.4). Immune regulatory genes included chemokines, chemokine receptors, major histocompatibility complex-related genes, immunostimulatory molecules, immunoinhibitory molecules, cytokines, and immune checkpoint-related genes. Correlation results were visualized using R-generated heatmaps.

### 2.6. Immunotherapy Response Analysis

The association between P2RY13 expression and immunotherapy response was evaluated using publicly available immunotherapy-treated cohorts accessed through the ROC Plotter platform (https://rocplot.com/, accessed on 15 April 2026). Three independent cohorts were included, comprising patients treated with anti-MAGE-A3, anti-PD-1/CTLA-4, or anti-PD-1/PD-L1 therapies. Patients were classified as responders or non-responders according to the response annotations provided in the original datasets.

P2RY13 expression levels were compared between responders and non-responders. Receiver operating characteristic (ROC) curve analysis was performed to evaluate the predictive performance of P2RY13 expression for immunotherapy response. The area under the curve (AUC) was calculated for each cohort.

### 2.7. Single-Cell RNA-Sequencing Data Acquisition, Processing, and Macrophage Subset Definition

Single-cell RNA-sequencing (scRNA-seq) datasets for LUAD, LIHC, and CRC were obtained from the Tumor Immune Single-cell Hub 2 [23] (TISCH2; https://tisch.compbio.cn/home/, accessed on 15 April 2026), with priority given to datasets containing both tumor and matched normal tissues when available. Processed expression matrices and cell-type annotations were downloaded for downstream analyses. P2RY13 expression was evaluated in major cell populations, including epithelial/tumor cells, T cells, B cells, myeloid cells, endothelial cells, and fibroblasts. Given the association between P2RY13 expression and myeloid infiltration identified in bulk analyses, annotated macrophages were extracted for focused analysis and visualized using uniform manifold approximation and projection (UMAP). Macrophages were classified as P2RY13-positive or P2RY13-negative according to the presence or absence of detectable P2RY13 transcript expression, respectively. In tumor tissues, these subsets were considered TAM-related populations. The proportions of P2RY13- negative macrophages were calculated in tumor and normal tissues for each cancer type to evaluate relative changes during tumorigenesis.

Nevertheless, this detection-based classification has an inherent methodological limitation. Because scRNA-seq is characterized by sparse and incomplete transcript capture, failure to detect P2RY13 in an individual macrophage does not necessarily indicate the true absence of its expression. Therefore, we used the term “P2RY13-negative” as an operational, detection-based designation for macrophages with no detectable P2RY13 transcripts under the applied analytical conditions. Accordingly, the resulting proportions were interpreted as relative estimates of the transcript-undetected population rather than precise measurements of the true prevalence of biologically P2RY13-null macrophages.

### 2.8. Single-Cell-Derived P2RY13-Negative TAM and NET-Related Signature Analysis

Single-cell RNA-seq datasets from LUAD, LIHC, and CRC were analyzed separately to identify P2RY13-negative TAMs and derive transcriptional signatures. Within each cancer type, TAMs without detectable P2RY13 transcripts were compared with P2RY13-positive TAMs. Genes significantly upregulated in the P2RY13-negative subset were used to construct the corresponding P2RY13-negative TAM signature. The gene sets derived from the LUAD, LIHC, and CRC datasets are provided in Tables S1–S3, respectively.

The single-cell-derived signatures were subsequently projected onto bulk transcriptomic data from the TCGA-LUAD, TCGA-LIHC, and TCGA-COADREAD cohorts. For each patient, a P2RY13-negative TAM signature score was calculated using single-sample gene set enrichment analysis. NET-related signature scores were calculated independently using a previously reported gene set and scoring method [24]. Within each cancer cohort, the association between P2RY13-negative TAM and NET-related signature scores was evaluated using Spearman correlation analysis. These analyses were performed to assess the transcriptomic association between the relative enrichment of the P2RY13-negative TAM state and NET-related activity in bulk tumor tissues.

### 2.9. Tissue Microarray Acquisition

Tissue microarrays (TMAs) containing paired tumor and adjacent normal tissues from LUAD, LIHC, and CRC were purchased from Shanghai Outdo Biotech Co., Ltd. (Shanghai, China). The CRC TMA (Lot XT15-008, HCol-Ade060Lym-01) included 20 patients (60 cores), the LIHC TMA (Lot I11-003, HLiv-HCC050PG-01) included 25 patients (50 cores), and the LUAD TMA (Lot XT24-015, HLugA060PG03) included 30 patients (60 cores). Baseline clinicopathological characteristics are summarized in Tables S4–S6.

### 2.10. Immunohistochemistry

TMA sections were subjected to immunohistochemical staining. After deparaffinization, antigen retrieval was performed by microwave heating in 5 mM Tris-HCl buffer for 10 min. Endogenous peroxidase activity was blocked with 3% hydrogen peroxide, followed by serum blocking to minimize non-specific antibody binding. Sections were then incubated overnight at 4 °C with antibodies against citrullinated histone H3 (histone H3 [citrullinated at Arg2, Arg8, and Arg17]; 1:200; Novus Biologicals, Centennial, CO, USA) or P2RY13 (1:250; Proteintech, Wuhan, China). After incubation with horseradish peroxidase-conjugated secondary antibodies for 30 min at room temperature, immunoreactivity was visualized using diaminobenzidine, and sections were counterstained with hematoxylin.

### 2.11. Multiplex Immunofluorescence Staining and Quantitative Analysis

Multiplex immunofluorescence (mIF) staining was performed on TMA sections to identify and quantify P2RY13-negative TAMs and NETs. After deparaffinization and rehydration, sections underwent antigen retrieval, followed by blocking of endogenous peroxidase activity and non-specific antibody binding. Sections were sequentially incubated with antibodies against MPO (1:250; Proteintech, Wuhan, China), citrullinated histone H3 (histone H3 [citrullinated at Arg2, Arg8, and Arg17]; 1:200; Novus Biologicals, Centennial, CO, USA), P2RY13 (1:250; Proteintech, Wuhan, China), and CD68 (1:250; Proteintech, Wuhan, China). Multiplex detection was performed using a sequential tyramide signal amplification protocol, and nuclei were counterstained with DAPI to generate a four-marker, five-color mIF panel.

P2RY13^−^CD68^+^ cells were defined as P2RY13-negative macrophages, whereas NETs were identified according to established criteria as extracellular web-like structures exhibiting colocalized MPO, CitH3, and DAPI signals [25]. For quantitative analysis, three randomly selected, non-overlapping high-power fields (×400) within the tumor region of each TMA core were evaluated. P2RY13-negative TAMs were counted in each field, and the mean count per high-power field was used to represent the infiltration level. NET-associated areas were quantified, and NET expression was calculated as the percentage of the analyzed tumor area occupied by MPO^+^/CitH3^+^ extracellular web-like structures. The mean percentage from the three fields was recorded as the NET expression level for each sample.

### 2.12. Cell Culture and Macrophage-like Differentiation

THP-1 and U937 cells were maintained in RPMI 1640 medium supplemented with 10% fetal bovine serum, 100 IU/mL penicillin, and 100 μg/mL streptomycin at 37 °C in a humidified atmosphere containing 5% CO_2_. To generate M0-like macrophages, THP-1 and U937 cells were treated with phorbol 12-myristate 13-acetate (PMA; 100 ng/mL) for 48 h. The PMA-containing medium was then removed, and cells were washed with phosphate-buffered saline (PBS) and allowed to rest in fresh complete medium for an additional 24 h. Acquisition of an adherent macrophage-like morphology was used to confirm successful differentiation before treatment with tumor-cell-conditioned medium.

### 2.13. Generation of Tumor-Educated TAM-like Macrophages

A549, Hep3B, and SW480 cells were used to generate tumor-cell-conditioned medium (TCM) representative of lung cancer, hepatocellular carcinoma, and colorectal cancer, respectively. All cell lines were maintained in DMEM supplemented with 10% fetal bovine serum, 100 IU/mL penicillin, and 100 μg/mL streptomycin at 37 °C. When cultures reached approximately 80% confluence, cells were washed twice with PBS and incubated for an additional 24 h in DMEM containing 1% fetal bovine serum. Supernatants were then collected, centrifuged at 1000× *g* for 10 min at 4 °C, and passed through 0.22-μm filters to remove residual cells and debris. The resulting A549-, Hep3B-, and SW480-derived TCM was used immediately or stored in aliquots at −80 °C, avoiding repeated freeze–thaw cycles. To generate tumor-educated TAM-like macrophages, PMA-differentiated THP-1- and U937-derived M0-like macrophages were exposed for 48 h to the corresponding TCM mixed 1:1 (*v*/*v*) with fresh complete RPMI 1640 medium.

### 2.14. P2RY13 Knockdown and Rescue

After tumor education, THP-1- and U937-derived TAM-like macrophages were washed with PBS and assigned to one of three groups: negative-control siRNA (siNC), siRNA targeting P2RY13 (siP2RY13), or siP2RY13 plus a P2RY13 overexpression construct (OE-P2RY13) for rescue. Cells were transfected using Lipofectamine 2000(Thermo Fisher Scientific, Waltham, MA, USA) according to the manufacturer’s instructions, with siRNAs used at a final concentration of 50 nM. P2RY13-targeting and negative-control siRNAs were purchased from RiboBio Co., Ltd. (Guangzhou, China), whereas the P2RY13 overexpression construct was generated by GeneChem Co., Ltd. (Shanghai, China). P2RY13 protein expression was assessed by western blotting to confirm knockdown and rescue efficiencies.

### 2.15. Neutrophil Isolation and Conditioned-Medium Treatment

Primary human neutrophils were isolated from peripheral blood samples obtained from healthy donors using Polymorphprep density-gradient medium (Axis-Shield, Dundee, UK) according to the manufacturer’s instructions. Neutrophil purity was assessed using a Fast Giemsa Staining Kit (Yeasen Biotechnology, Shanghai, China), after which freshly isolated cells were resuspended in RPMI 1640 medium (Gibco, Grand Island, NY, USA). Conditioned medium was collected separately from each experimental group of tumor-educated TAM-like macrophages and applied to freshly isolated neutrophils for 16 h. This conditioned-medium culture system was used to evaluate the effects of soluble factors released by tumor-educated TAM-like macrophages on neutrophils.

### 2.16. In Vitro Assessment of NET Formation

Following conditioned-medium treatment, neutrophils cultured on coverslips were processed for immunofluorescence staining. Briefly, cells were fixed with 4% paraformaldehyde for 30 min, permeabilized with 0.5% Triton X-100 for 15 min, and blocked with 1% bovine serum albumin in PBS for 1 h. Primary antibodies against citrullinated histone H3 (histone H3 [citrullinated at Arg2, Arg8, and Arg17]; 1:200; Novus Biologicals) and MPO (5 μg/mL; R&D Systems) were applied overnight at 4 °C. After incubation with the corresponding Alexa Fluor-conjugated secondary antibodies for 1 h at room temperature, nuclei and extracellular DNA were stained with DAPI (Sigma-Aldrich, USA) for 5 min. Fluorescence images were subsequently acquired using a fluorescence microscope.

In parallel, culture supernatants were collected after conditioned-medium treatment and analyzed for CitH3 using a commercially available ELISA kit (Cayman Chemical, Ann Arbor, MI, USA) according to the manufacturer’s instructions. CitH3 concentrations were determined from standard curves fitted using a four-parameter logistic regression model in GraphPad Prism (version 11.0.1).

### 2.17. Western Blotting

Western blotting was performed to determine P2RY13 protein expression and verify the efficiencies of P2RY13 knockdown and subsequent re-expression. Protein lysates from the indicated experimental groups were subjected to immunoblotting using antibodies against P2RY13 (1:2000; Proteintech, Wuhan, China) and β-actin (1:20,000; Proteintech, Wuhan, China). β-Actin served as the loading control, and P2RY13 protein expression was normalized to β-actin.

### 2.18. Quantification of Neutrophil Infiltration in TMA

Neutrophil infiltration was evaluated in hematoxylin and eosin-stained TMA sections from LUAD, LIHC, and CRC tissues. Each tissue core was initially examined at low magnification to identify viable tumor regions suitable for evaluation, and areas containing necrosis, hemorrhage, tissue folds, uneven staining, or other processing artifacts were excluded. Three non-overlapping high-power fields (×400) were subsequently selected from each evaluable core. Neutrophils were identified based on their characteristic histomorphological features, including deeply stained segmented or multilobed nuclei and scant, lightly eosinophilic granular cytoplasm. Only morphologically intact neutrophils within the tumor parenchyma or stroma were counted. Cells within vascular lumina, nuclear debris in necrotic regions, and inflammatory cells that could not be reliably identified morphologically were excluded. Neutrophil infiltration was expressed as the mean number of neutrophils per high-power field and subsequently correlated with the infiltration level of P2RY13^−^CD68^+^ TAMs in the corresponding tumor tissue.

### 2.19. Statistical Analysis

Statistical analyses were performed using R (version 3.6.4) and GraphPad Prism (version 11.0.1) according to the characteristics of the data. Student’s *t*-test was used for data with equal variance, whereas the Mann–Whitney U test was applied to data with unequal variance. For comparisons involving small sample sizes, differences between two independent groups were assessed using a two-sided Welch’s *t*-test without assuming equalvariances. Pearson correlation analysis was used to evaluate correlations, and the log-rank test was applied for survival analysis. A *p* value < 0.05 was considered statistically significant. Schematic diagrams were generated using BioGDP.com.

## 3. Results

### 3.1. P2RY13 Is Downregulated in Multiple Cancer Types and Associated with Unfavorable Prognosis

**Figure 1:**
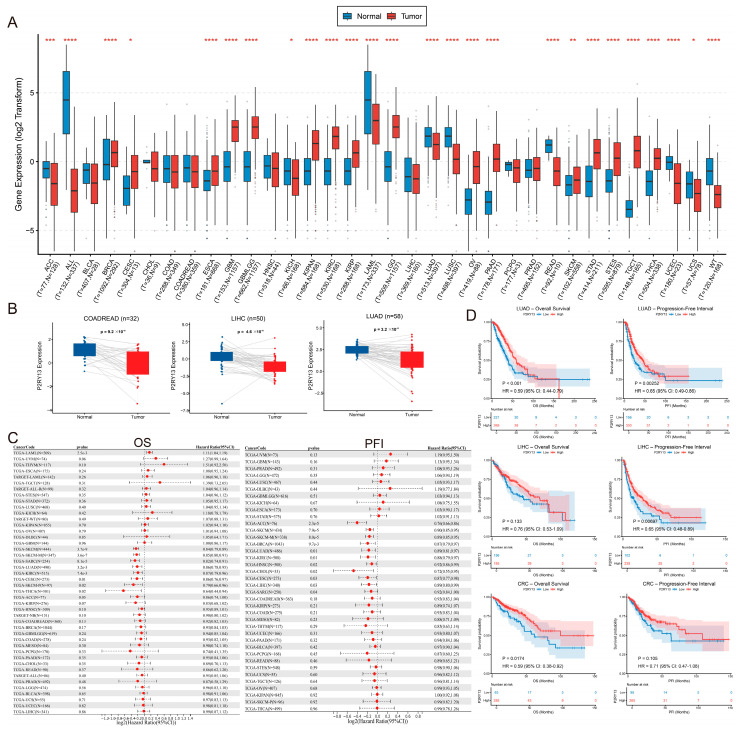
Pan-cancer expression pattern and prognostic significance of P2RY13. (**A**) Pan-cancer comparison of P2RY13 expression between tumor and normal tissues based on TCGA and GTEx datasets. The *y*-axis represents log2-transformed gene expression levels, while the *x*-axis indicates different cancer types. Box plots show the distribution of P2RY13 expression in tumor and normal tissues among cancers. (**B**) Paired comparison of P2RY13 expression between tumor and adjacent normal tissues in the COADREAD, LIHC, and LUAD cohorts. (**C**) Pan-cancer Cox proportional hazards regression analyses showing associations between P2RY13 expression and overall survival (OS) or progression-free interval (PFI). Forest plots present hazard ratios (HRs) and 95% confidence intervals (CIs) for each cancer type. An HR < 1 indicates that higher P2RY13 expression is associated with a lower risk of death or disease progression. (**D**) Kaplan–Meier survival curves for OS and PFI according to P2RY13 expression in LUAD, LIHC, and the COADREAD cohort. Patients were stratified into high- and low-expression groups based on P2RY13 expression levels. Curves illustrate differences in survival probability between groups across survival endpoints. *p* values, HRs, and 95% CIs are shown in the figure. Asterisks indicate statistical significance: * *p* < 0.05, ** *p* < 0.01, *** *p* < 0.001, and **** *p* < 0.0001.

Transcriptomic analysis of TCGA/GTEx datasets revealed heterogeneous P2RY13 expression among cancers, with an overall trend toward downregulation in multiple solid tumors, particularly LUAD, LIHC, and CRC (Figure 1A). Paired tumor–normal analyses further confirmed significantly reduced P2RY13 expression in LUAD (*p* = 3.2 × 10^−8^), LIHC (*p* = 4.6 × 10^−6^), and COADREAD (*p* = 9.2 × 10^−6^). Similar downregulation was observed in BRCA but not in STAD (Figure 1B and Figure S1). Cox regression and Kaplan–Meier survival analyses further demonstrated that higher P2RY13 expression was associated with improved OS, PFI, disease-specific survival, and disease-free interval in these three tumor types (Figure 1C,D and Figures S2 and S3). Collectively, these findings indicate that reduced P2RY13 expression is closely associated with poor clinical outcomes, identifying P2RY13 downregulation as a potential adverse prognostic factor, particularly in LUAD, LIHC, and CRC.

### 3.2. P2RY13 Correlates with an Immune-Inflamed Tumor Microenvironment and Reduced Expression Is Associated with Impaired Immunotherapy Response

**Figure 2:**
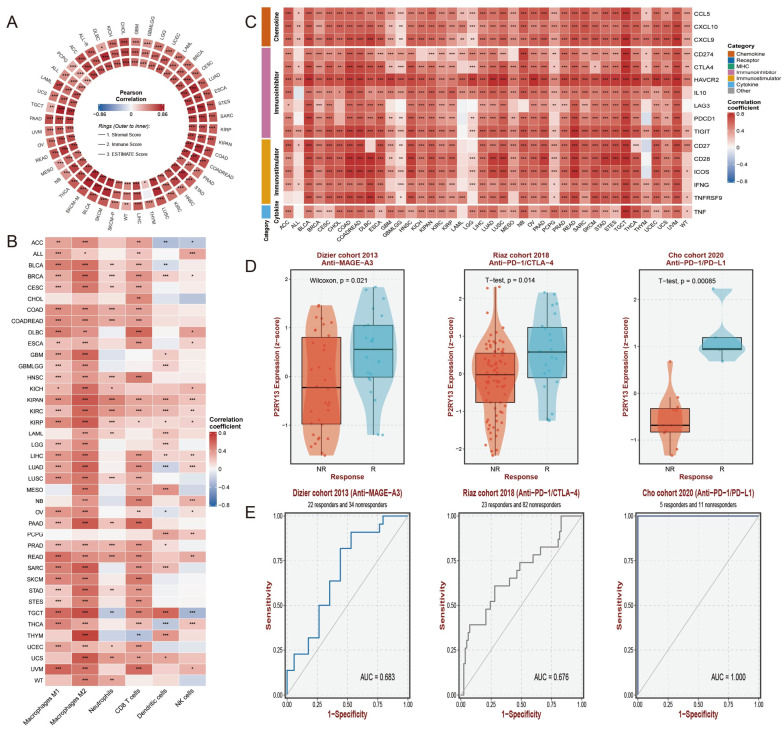
P2RY13 expression is associated with an immune-inflamed tumor microenvironment and predicts immunotherapy response. (**A**) P2RY13 expression positively correlates with Stromal Score, Immune Score, and ESTIMATE Score in pan-cancer cohorts (Pearson r = 0.86, *p* < 0.001), indicating enhanced stromal and immune infiltration in P2RY13-high tumors. (**B**) quanTIseq analysis reveals positive correlations between P2RY13 expression and key immune populations, particularly macrophages and neutrophils (|r| > 0.4, *p* < 0.01), highlighting their contribution to the P2RY13-associated immune phenotype. (**C**) P2RY13 co-expression is observed with a broad spectrum of immune regulators, including chemokines (e.g., CCL5, CXCL9, and CXCL10), immunostimulatory molecules (e.g., CD27, CD28, and IFNG), and immune checkpoint genes (e.g., CD274 [PD-L1], PDCD1 [PD-1], CTLA4, and LAG3) (r > 0.3, *p* < 0.01), consistent with an activated, yet checkpoint-regulated, immune microenvironment. (**D**) In three independent immunotherapy cohorts [26], P2RY13 expression is significantly higher in responders (Rs) than in non-responders (NRs) (Dizier: Wilcoxon *p* = 0.021; Riaz: *t*-test *p* = 0.014; Cho: *t*-test *p* = 0.00085). (**E**) ROC analysis demonstrates the predictive performance of P2RY13 for immune checkpoint blockade response (Dizier AUC = 0.683; Riaz AUC = 0.676; Cho AUC = 1.000), supporting its potential utility as a companion diagnostic biomarker. Asterisks indicate statistical significance: * *p* < 0.05, ** *p* < 0.01, *** *p* < 0.001.

Given the pan-cancer downregulation of P2RY13 and its association with poor clinical outcomes, its potential relationship with TIME remodeling was investigated. ESTIMATE analysis revealed that P2RY13 expression was positively correlated with Stromal Score, Immune Score, and ESTIMATE Score in multiple tumor types (Figure 2A), suggesting that P2RY13-high tumors exhibit enhanced stromal and immune infiltration. Immune deconvolution using quanTIseq further demonstrated positive associations between P2RY13 expression and several immune cell populations, including CD8^+^ T cells, natural killer cells, dendritic cells, macrophages, and neutrophils (Figure 2B). These findings were validated using MCPcounter and EPIC, which consistently confirmed correlations with monocytic/macrophage-lineage cells, neutrophils, CD8^+^ T cells, and additional immune subsets (Figures S6 and S7). Collectively, these results indicate that P2RY13 expression reflects a broadly immune-infiltrated tumor state rather than a platform-specific association.

The relationship between P2RY13 expression and immune regulatory networks was subsequently examined. P2RY13 expression showed positive correlations with multiple chemokines and immune effector molecules, including CCL5, CXCL9, CXCL10, CD27, CD28, and IFNG. Positive associations were also observed with immune checkpoint and inhibitory molecules, including CD274 (PD-L1), PDCD1 (PD-1), CTLA4, and LAG3 (Figure 2C). Broader pan-cancer profiling of immune regulatory genes supported this pattern, indicating the coexistence of immune activation and compensatory inhibitory signaling (Figure S4). Immunophenoscore analysis further demonstrated positive correlations between P2RY13 expression and major histocompatibility complex-related and effector-cell scores (Figure S5), whereas negative correlations with suppressor-cell and checkpoint scores were observed in several tumor types. These findings suggest that P2RY13-high tumors are characterized by enhanced antigen presentation, increased immune effector activity, and an activated, yet feedback-regulated, immune microenvironment.

The potential relevance of P2RY13 in the immunotherapy response was further evaluated (Figure 2D,E). Analysis of three independent immunotherapy-treated cohorts from the ROC Plotter platform, including patients receiving anti-MAGE-A3, anti-PD-1/CTLA-4, or anti-PD-1/PD-L1 therapy, demonstrated significantly higher P2RY13 expression in responders than in non-responders. ROC analysis further supported the predictive value of P2RY13, yielding AUC values of 0.683, 0.676, and 1.000 in the Dizier, Riaz, and Cho cohorts, respectively. Although interpretation of the Cho cohort is limited by its small sample size, the consistent association observed among independent datasets supports P2RY13 as a candidate biomarker of immunotherapy responsiveness.

Together, these findings indicate that P2RY13 is associated with an immune-inflamed TIME and provide a rationale for subsequent single-cell analyses focused on its cellular origin and functional role within myeloid populations, particularly macrophages and neutrophils.

### 3.3. P2RY13-Negative TAMs Predominantly Infiltrate Tumor Tissues and Are Positively Associated with NET-Related Signatures

**Figure 3:**
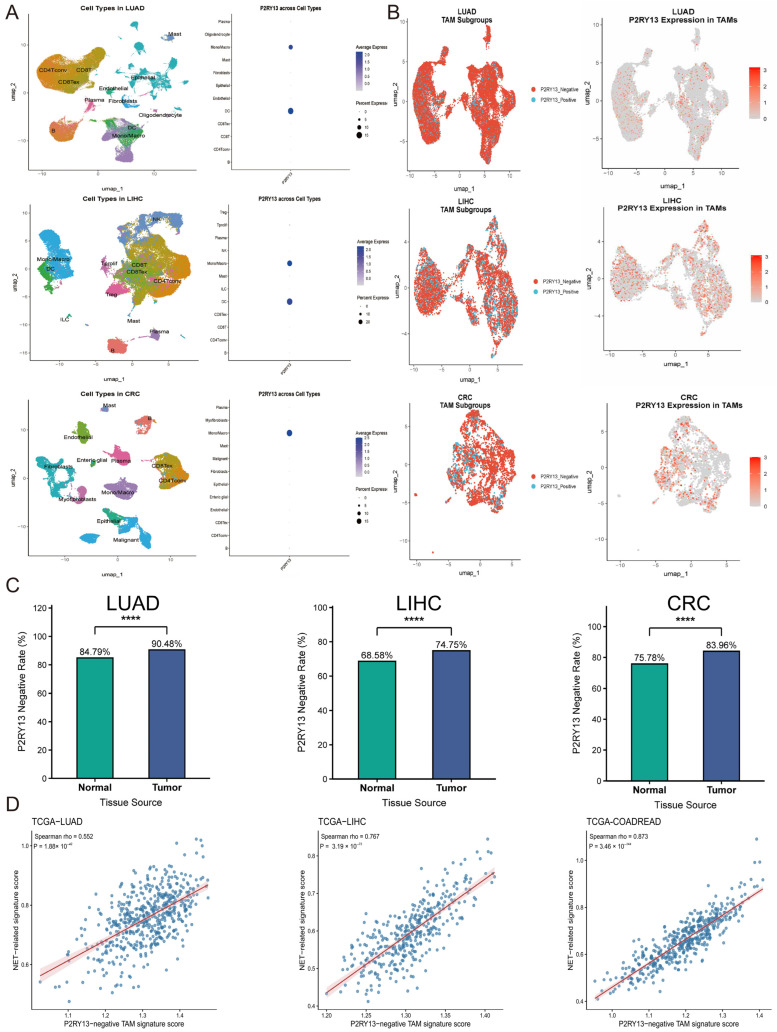
Single-cell transcriptomic analysis identifies P2RY13-negative TAMs associated with elevated NET-related programs. (**A**,**B**) Single-cell transcriptomic analysis of LUAD, LIHC, and CRC datasets showing P2RY13 expression in major cell populations and within the macrophage compartment. (**C**) Quantification of the proportion of P2RY13-negative macrophages in normal and tumor tissues in LUAD, LIHC, and CRC. The P2RY13-negative rate increased from 84.79% to 90.48% in LUAD, from 68.58% to 74.75% in LIHC, and from 75.78% to 83.96% in CRC. (**D**) Spearman correlation analyses between P2RY13-negative TAM signature scores and NET-related signature scores in the TCGA-LUAD, TCGA-LIHC, and TCGA-COADREAD cohorts. Spearman correlation coefficients and *p* values are shown. Asterisks indicate statistical significance: **** *p* < 0.0001.

To define the cellular context of P2RY13 expression within the tumor microenvironment, single-cell RNA-sequencing datasets from LUAD, LIHC, and CRC were analyzed. Following quality control, dimensionality reduction, and cell-type annotation, major cellular compartments were identified, including epithelial/tumor, lymphoid, myeloid, endothelial, and fibroblast populations. Consistent with the pan-cancer immune infiltration analyses, P2RY13 expression was predominantly detected in macrophages and exhibited marked intercellular heterogeneity, supporting stratification into P2RY13-positive and P2RY13-negative macrophage subsets for subsequent analyses (Figure 3A,B).

Quantitative comparison of tumor and normal tissues showed that the proportion of P2RY13-negative macrophages—operationally defined as macrophages without detectable P2RY13 transcripts—was higher in tumor tissues. Specifically, the proportion of P2RY13-negative macrophages increased from 84.79% to 90.48% in LUAD, from 68.58% to 74.75% in LIHC, and from 75.78% to 83.96% in CRC (Figure 3C).

Projection of the single-cell-derived signatures onto the corresponding TCGA bulk transcriptomic datasets revealed significant positive correlations between P2RY13-negative TAM signature scores and NET-related signature scores in LUAD, LIHC, and CRC (Figure 3D). Thus, tumors enriched for the P2RY13-negative TAM transcriptional state also exhibited greater NET-related transcriptional activity.

### 3.4. Tissue Microarray Validation Demonstrates the Enrichment of P2RY13-Negative TAMs in LUAD, LIHC, and CRC

**Figure 4:**
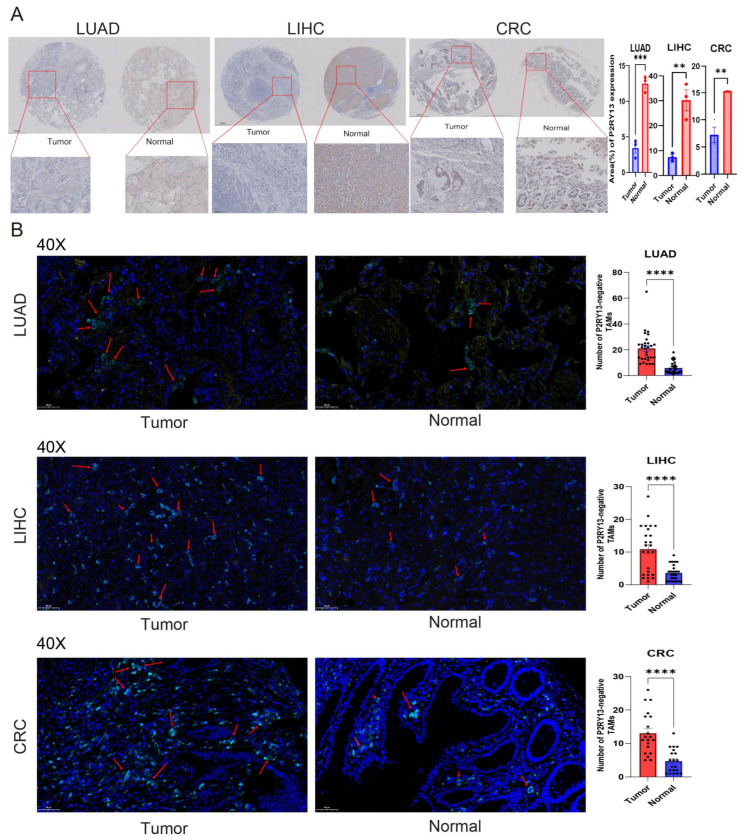
Tissue microarray validation of reduced P2RY13 expression and increased infiltration of P2RY13-negative macrophages in LUAD, LIHC, and CRC. (**A**) Representative immunohistochemical staining images of P2RY13 in tumor tissues and corresponding adjacent normal tissues from LUAD, LIHC, and CRC tissue microarrays. Compared with adjacent normal tissues, tumor tissues exhibited reduced P2RY13 immunoreactivity in all three cancer types. (**B**) Representative multiplex immunofluorescence images and quantitative analysis of P2RY13-negative macrophage infiltration in tumor tissues and paired adjacent normal tissues. Macrophages were identified by CD68 positivity (cyan), and P2RY13 was visualized in yellow. CD68^+^ cells lacking detectable P2RY13 staining were classified as P2RY13-negative macrophages, and arrows in the images mark typical P2RY13(-) macrophages Infiltration of P2RY13-negative macrophages was significantly greater in tumor tissues than in paired adjacent normal tissues across all three cancer types. Asterisks indicate statistical significance: ** *p* < 0.01, *** *p* < 0.001, and **** *p* < 0.0001.

To validate observations derived from transcriptomic and single-cell analyses, immunohistochemical staining was performed on tissue microarrays containing three common solid tumor types: LUAD, LIHC, and CRC. Paired tumor and adjacent normal tissues were included for each tissue microarray section. The CRC cohort comprised 20 patients and 60 cores, the LIHC cohort comprised 25 patients and 50 cores, and the LUAD cohort comprised 30 patients and 60 cores. Immunohistochemical analyses demonstrated markedly reduced P2RY13 staining intensity in tumor tissues compared with adjacent normal tissues for LUAD, LIHC, and CRC (Figure 4A).

mIF staining was subsequently performed to identify P2RY13-negative TAMs. Cells exhibiting a P2RY13^−^CD68^+^ phenotype were classified as P2RY13-negative TAMs. Quantitative analysis demonstrated significantly greater infiltration of P2RY13-negative TAMs in tumor tissues than in the corresponding adjacent normal tissues in all three cohorts (Figure 4B). These findings were consistent with the transcriptomic and single-cell analyses, further supporting enrichment of P2RY13-negative TAMs within the tumor microenvironment.

### 3.5. P2RY13-Silenced TAM-like Macrophages Promote Neutrophil NETosis

**Figure 5:**
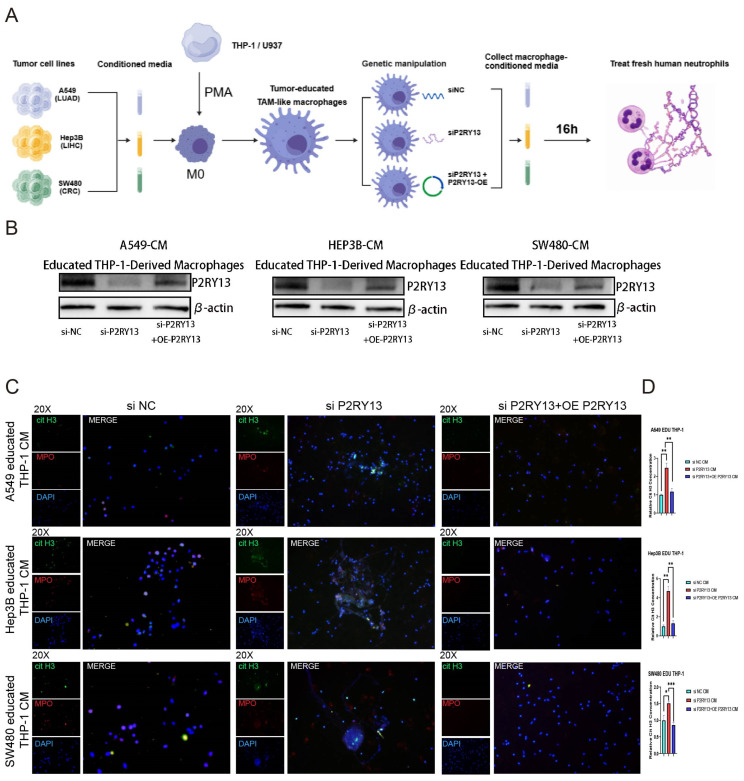
P2RY13 knockdown enhances the NET-promoting paracrine activity of tumor-educated TAM-like macrophages. (**A**) Schematic of the conditioned-medium transfer system. PMA-differentiated THP-1- and U937-derived macrophages were educated with tumor-cell-conditioned medium from A549, Hep3B, or SW480 cells to generate TAM-like macrophages representative of the LUAD, LIHC, and CRC microenvironments, respectively. Following P2RY13 knockdown or rescue, macrophage-conditioned medium was applied to freshly isolated human neutrophils. (**B**) Western blot analysis of P2RY13 expression in THP-1-derived TAM-like macrophages transfected with siNC, siP2RY13, or siP2RY13 plus the OE-P2RY13 rescue construct. β-Actin served as the loading control. (**C**) Representative immunofluorescence images showing NET formation in neutrophils treated with conditioned medium from the indicated THP-1-derived TAM-like macrophage groups. Conditioned medium from P2RY13-silenced macrophages increased MPO^+^/CitH3^+^ extracellular DNA structures, whereas P2RY13 re-expression attenuated this effect. (**D**) ELISA quantification of CitH3 in neutrophil culture supernatants. CitH3 levels increased following treatment with conditioned medium from P2RY13-silenced macrophages and decreased after P2RY13 re-expression. Similar findings were observed in the corresponding U937-derived models (Figures S8–S10). Asterisks indicate statistical significance: * *p* < 0.05, ** *p* < 0.01, *** *p* < 0.001. The original image of western blot are shown in Supplementary File S1.

To determine whether P2RY13 downregulation in macrophages affects neutrophil NET formation, a conditioned-medium transfer system was established using THP-1- and U937-derived tumor-educated TAM-like macrophages (Figure 5A). PMA-differentiated macrophages were educated with TCM from A549, Hep3B, or SW480 cells to model the LUAD, LIHC, and CRC microenvironments, respectively. Following P2RY13 knockdown or rescue, macrophage-conditioned medium was collected and applied to freshly isolated human neutrophils. Knockdown and re-expression efficiencies were confirmed by western blotting (Figure 5B and Figure S8). Conditioned medium from P2RY13-silenced TAM-like macrophages significantly enhanced NET formation in the A549-, Hep3B-, and SW480-educated models, while P2RY13 re-expression significantly attenuated this effect (Figure 5C and Figure S9).

Consistently, ELISA demonstrated increased CitH3 levels in supernatants from neutrophils treated with conditioned medium from P2RY13-silenced TAM-like macrophages, whereas this increase was reversed by P2RY13 re-expression (Figure 5D and Figure S10). Collectively, these findings indicate that P2RY13 downregulation enhances the paracrine capacity of tumor-educated TAM-like macrophages to promote NET formation.

### 3.6. Tissue Microarray Analysis Reveals a Positive Correlation Between P2RY13-Negative TAM Infiltration and NET Formation in LUAD, LIHC, and CRC

**Figure 6:**
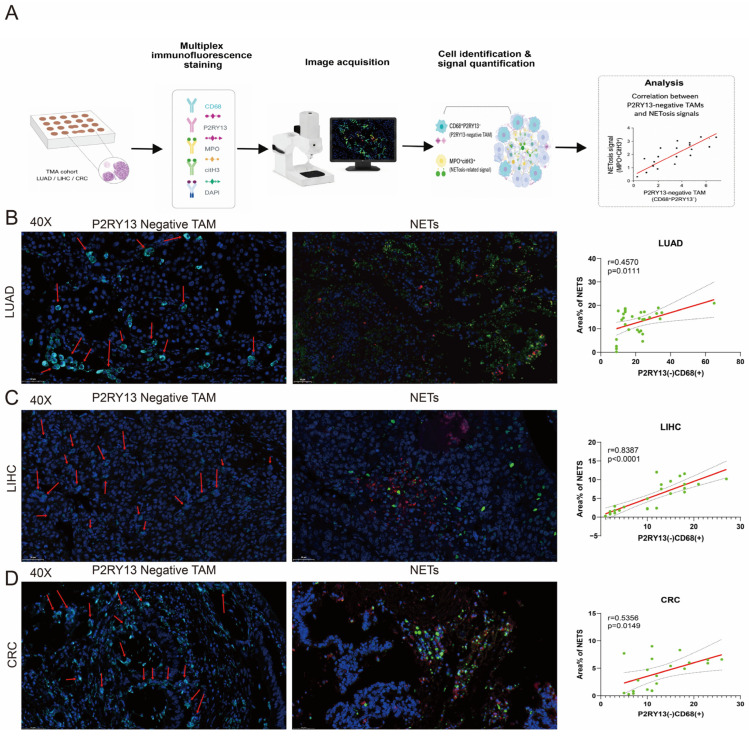
P2RY13-negative TAM infiltration positively correlates with NET formation in LUAD, LIHC, and CRC tissue microarrays. (**A**) Schematic overview of the histological quantification and correlation workflow. P2RY13^−^CD68^+^ cells were classified as P2RY13-negative TAMs. NETs were identified as extracellular web-like structures exhibiting colocalized MPO (red), CitH3 (green), and DAPI signals, and the arrows in immunofluorescence images mark typical P2RY13(-)TAM. (**B**–**D**) Spearman correlation analyses between P2RY13-negative TAM infiltration and NET expression, quantified as the area fraction occupied by MPO^+^/CitH3^+^ extracellular structures, in LUAD (**B**), LIHC (**C**), and CRC (**D**). Greater infiltration of P2RY13-negative TAMs was significantly associated with higher NET expression across all three cancer types. Spearman correlation coefficients (r) and *p* values are shown.

To examine the relationship between P2RY13-negative TAMs and NET formation in tumor tissues, P2RY13^−^CD68^+^ TAM infiltration and NET expression were quantified by mIF, whereas neutrophil infiltration was assessed in matched hematoxylin and eosin-stained sections. The histological quantification and correlation workflow is summarized in Figure 6A.

Across the LUAD, LIHC, and CRC cohorts, tumors with greater P2RY13-negative TAM infiltration exhibited higher NET expression, defined as the area fraction occupied by MPO^+^/CitH3^+^ extracellular web-like structures. Spearman correlation analysis confirmed significant positive correlations between P2RY13-negative TAM infiltration and NET expression in LUAD (r = 0.4570, *p* = 0.0111; Figure 6B), LIHC (r = 0.8387, *p* <0.0001; Figure 6C), and CRC (r = 0.5356, *p* = 0.0149; Figure 6D). P2RY13-negative TAM infiltration was also positively correlated with histomorphologically quantified neutrophil infiltration across all three cancer types (Figure S11). Collectively, these findings link enrichment of P2RY13-negative TAMs with increased neutrophil infiltration and intratumoral NET formation.

### 3.7. P2RY13-Negative TAMs Exhibit Conserved Inflammatory and Chemotactic Transcriptional Features in LUAD, LIHC, and CRC

**Figure 7:**
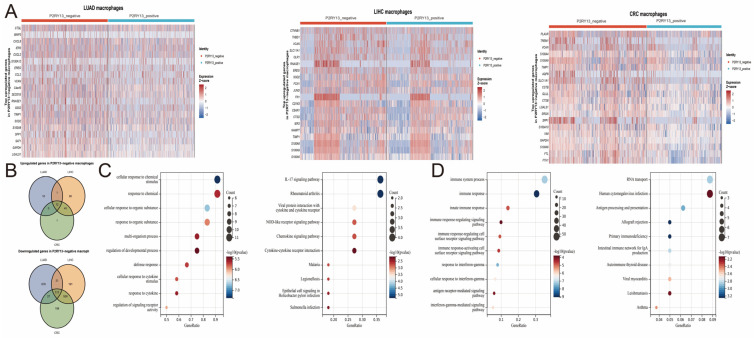
Conserved transcriptional features of P2RY13-negative TAMs across LUAD, LIHC, and CRC. (**A**) Heatmaps showing representative genes upregulated in P2RY13-negative TAMs relative to P2RY13-positive TAMs. (**B**) Venn diagrams showing 12 commonly upregulated and 171 commonly downregulated genes across the three cancer types. (**C**) GO and KEGG enrichment analyses of the commonly upregulated genes, including inflammatory and cytokine responses, chemokine signaling, IL-17 signaling, and NOD-like receptor signaling. (**D**) GO and KEGG enrichment analyses of the commonly downregulated genes, including immune response, interferon-γ response, antigen receptor-mediated signaling, and antigen processing and presentation. Dot size represents gene count, and color represents −log_10_ (*p* value).

Differential expression analysis was performed between P2RY13-negative and P2RY13-positive TAMs in single-cell RNA-seq datasets from LUAD, LIHC, and CRC. Compared with P2RY13-positive TAMs, P2RY13-negative TAMs exhibited increased expression of inflammation-, chemotaxis-, and myeloid activation-related genes, including *SPP1*, *S100A8*, *S100A9*, *TIMP1*, *VCAN*, *CIDEC*, *CXCL2*, *CXCL8*, *EREG*, and *LGALS1* (Figure 7A). Intersection analysis identified 12 commonly upregulated and 171 commonly downregulated genes across the three cancer types (Figure 7B).

The commonly upregulated genes were enriched in Gene Ontology (GO) terms related to chemical and cytokine responses and defense responses. Kyoto Encyclopedia of Genes and Genomes (KEGG) analysis revealed enrichment of the IL-17 signaling pathway, NOD-like receptor signaling pathway, chemokine signaling pathway, and cytokine–cytokine receptor interaction (Figure 7C).

The commonly downregulated genes were enriched in GO terms associated with immune response, interferon-γ response, and antigen receptor-mediated signaling. KEGG analysis further identified enrichment of antigen processing and presentation, primary immunodeficiency, the intestinal immune network for IgA production, and allograft rejection (Figure 7D).

## 4. Discussion

This study characterizes P2RY13-negative TAMs as a tumor-enriched macrophage state associated with neutrophil infiltration and NET-related features and provides experimental evidence that P2RY13-dependent alterations in macrophage-conditioned medium modulate NET formation in vitro. At the pan-cancer level, P2RY13 was frequently downregulated in solid tumors and showed marked reductions in LUAD, LIHC, and COADREAD/CRC, where low expression was associated with poorer clinical outcomes (Figure 1 and Figures S1–S3). P2RY13 expression was also associated with an immune-inflamed tumor microenvironment and was higher in responders across three exploratory immunotherapy cohorts, although its predictive value requires validation in larger prospective cohorts (Figure 2 and Figures S4–S7). Single-cell analyses further revealed an increased proportion of macrophages lacking detectable P2RY13 transcripts in tumor tissues. Cancer-specific signatures derived from these cells were positively correlated with NET-related signatures after projection onto the corresponding TCGA bulk transcriptomic cohorts (Figure 3). Protein-level analyses demonstrated reduced P2RY13 expression and increased infiltration of P2RY13^−^CD68^+^ macrophages in LUAD, LIHC, and CRC tumors (Figure 4). Conditioned medium from P2RY13-silenced, tumor-educated TAM-like macrophages enhanced NET formation, whereas P2RY13 re-expression attenuated this effect (Figure 5). In parallel, tissue analyses demonstrated positive associations between P2RY13-negative TAM infiltration and both NET expression and neutrophil infiltration (Figure 6 and Figure S11). Collectively, these complementary findings support an association between the P2RY13-negative TAM state and a neutrophil- and NET-enriched tumor microenvironment but do not, by themselves, establish a direct causal mechanism in vivo.

P2RY13 belongs to the purinergic receptor family, which is involved in extracellular nucleotide sensing, inflammatory regulation, and immune cell communication [27,28]. Purinergic signaling is increasingly recognized as an important regulatory axis in cancer, particularly within the tumor microenvironment, where ATP, ADP, adenosine, and related metabolites shape immune cell recruitment, activation, and suppression. Consistent with these observations, pan-cancer and single-cell analyses demonstrated strong associations between P2RY13 expression and immune components of the tumor microenvironment. Notably, single-cell transcriptomic analyses showed minimal P2RY13 expression in malignant epithelial cells but predominant localization within macrophage populations (Figure 3A). This distribution suggests that the tumor-associated functions of P2RY13 may be mediated primarily through immune stromal components, particularly macrophages, rather than through tumor-intrinsic mechanisms. Importantly, the positive association between bulk tumor P2RY13 expression and immune infiltration does not necessarily conflict with the enrichment of P2RY13-negative macrophages in tumors. Bulk P2RY13 expression reflects both immune cell abundance and gene expression across multiple cellular populations, whereas single-cell analysis specifically captures P2RY13 heterogeneity within the macrophage compartment. Accordingly, bulk-tissue P2RY13 expression should not be interpreted as a direct measure of macrophage-intrinsic P2RY13 activity or the abundance of P2RY13-negative TAMs.

Macrophages are highly plastic components of the tumor microenvironment and can adopt diverse functional states depending on tissue context [29,30,31]. The transcriptional profile identified in Figure 7 did not conform to the conventional M1/M2 polarization paradigm. P2RY13-negative TAMs exhibited enhanced inflammatory and chemotactic programs, including IL-17, NOD-like receptor, cytokine, and chemokine signaling, while showing relative attenuation of interferon-γ-response and antigen presentation pathways. This combination suggests a context-dependent, transcriptionally remodeled macrophage state rather than a uniformly M1- or M2-polarized phenotype. This interpretation is consistent with transcriptomic and single-cell studies showing that macrophage activation occurs along a continuum and that TAMs may simultaneously express features traditionally assigned to different polarization states [32,33]. Accordingly, tumor-cell-conditioned medium was used to generate tumor-educated TAM-like macrophages rather than imposing a defined IL-4/IL-13- or IFN-γ/LPS-induced polarization state. This approach exposes macrophages to soluble factors derived from three tumor-cell contexts and may better reflect mixed tumor-driven activation. Nevertheless, it does not fully recapitulate the cellular, extracellular matrix, metabolic, and spatial cues encountered by bona fide TAMs in vivo.

The association between P2RY13-negative TAMs and NET-related activity was supported at multiple analytical levels, although each has distinct interpretive limitations. Projection of the single-cell-derived signatures onto TCGA data demonstrated coordinated enrichment of P2RY13-negative TAM and NET-related transcriptional programs (Figure 3D), but these bulk-tissue scores do not directly quantify either cell abundance or NET formation. In the tissue microarrays, P2RY13-negative TAM infiltration was positively correlated with mIF-defined NET expression (Figure 6) and with histomorphologically quantified neutrophil infiltration (Figure S11). These findings suggest two non-mutually exclusive mechanisms. P2RY13-negative TAM-associated signals may promote neutrophil recruitment or retention within tumors, thereby increasing the cellular substrate available for NET formation, and/or enhance the propensity of infiltrating neutrophils to undergo NETosis. The current data do not demonstrate that macrophage P2RY13 directly suppresses NETosis. Rather, they suggest that preserved P2RY13 expression restrains a macrophage state whose paracrine activity promotes neutrophil accumulation and NET formation.

More importantly, the conditioned-medium experiments provide evidence of a P2RY13-dependent paracrine effect. Conditioned medium from P2RY13-silenced, tumor-educated TAM-like macrophages increased MPO^+^/CitH3^+^ extracellular DNA structures and CitH3 release by neutrophils, whereas P2RY13 re-expression attenuated these effects (Figure 5 and Figures S8–S10). Because macrophages and neutrophils were physically separated in this system, the observed phenotype is attributable to alterations in macrophage-conditioned medium rather than direct cell–cell contact. However, knockdown of a single receptor may affect multiple downstream pathways and secreted factors, and the present experiments do not identify the mediator responsible for the NET-promoting effect. The enrichment of CXCL2, CXCL8, S100A8, and S100A9 transcripts in P2RY13-negative TAMs, together with the chemokine-related enrichment observed in Figure 7, identifies candidate mechanisms for future investigation but does not demonstrate that these molecules were secreted or mediated the observed phenotype. Future studies should combine secretome profiling or cytokine arrays with targeted ELISA, neutralizing antibodies, receptor blockade, and recombinant-protein experiments to determine whether specific chemokines, cytokines, metabolites, or extracellular vesicles account for the effect. Validation using primary human TAMs and in vivo models will also be required to determine whether this paracrine interaction occurs within native tumor tissues.

Despite the potential significance of these findings, several limitations should be acknowledged. First, the designation “P2RY13-negative” was based on the absence of detectable P2RY13 transcripts in scRNA-seq data. Because single-cell transcript detection is sparse and low-abundance transcripts may escape detection, a zero value does not confirm complete biological absence of P2RY13 and may overestimate the P2RY13-negative fraction. Although a binary presence/absence criterion was adopted as a transparent operational threshold, the robustness of the findings to alternative expression thresholds was not assessed. Accordingly, the reported population should be interpreted as macrophages lacking detectable P2RY13 transcripts under the applied analytical conditions. Furthermore, transcript-defined P2RY13-negative macrophages and protein-defined P2RY13^−^CD68^+^ macrophages represent related, but not necessarily identical, populations. Second, the P2RY13-negative TAM and NET-related scores derived from bulk TCGA transcriptomes reflect the relative enrichment of their respective transcriptional programs rather than absolute P2RY13-negative TAM abundance or direct NET formation. Their correlation may also be influenced by differences in tumor cellular composition or by overlapping genes between the two signatures. Third, THP-1- and U937-derived tumor-educated macrophages are reductionist cell-line models and were not systematically benchmarked against the transcriptomic or proteomic profiles of primary human TAMs. Accordingly, their designation as TAM-like macrophages should be regarded as operational. In addition, canonical M1- and M2-associated markers were not evaluated in the TMA specimens. Therefore, although single-cell analysis suggests a mixed inflammatory and immune-remodeled phenotype, the relationship between P2RY13-negative TAMs and conventional macrophage polarization states remains to be validated at the protein and spatial levels. Fourth, the positive tissue correlation between P2RY13-negative TAMs and neutrophil counts does not demonstrate enhanced neutrophil recruitment because macrophage-conditioned neutrophil migration assays and in vivo trafficking studies were not performed. Moreover, hematoxylin and eosin-based neutrophil identification relies on histomorphological assessment. Fifth, the cytokines, chemokines, metabolites, extracellular vesicles, or other soluble mediators responsible for the NET-promoting activity of macrophage-conditioned medium remain undefined. Finally, the relatively small TMA cohorts, lack of comprehensive clinical follow-up data, and limited, heterogeneous immunotherapy cohorts constrain the prognostic and translational interpretation of these findings. Further validation using primary human TAMs, larger independent clinical cohorts, targeted secretome analyses, and in vivo models is warranted.

Despite these limitations, the findings identify a reproducible association between a P2RY13-low/undetectable macrophage state and neutrophil- and NET-related features across LUAD, LIHC, and CRC. The knockdown–rescue experiments further support a P2RY13-dependent alteration in the NET-promoting paracrine activity of tumor-educated TAM-like macrophages. These findings establish a testable macrophage–neutrophil interaction model that warrants mechanistic investigation rather than demonstrating a definitive causal pathway in patients.

## 5. Conclusions

This study suggests that P2RY13 is broadly downregulated in multiple cancers and may be associated with poor prognosis and potentially impaired immunotherapy responsiveness. P2RY13 is predominantly expressed in TAMs, whereas P2RY13-negative TAMs are enriched within tumor tissues in multiple cancer types, including LUAD, LIHC, and CRC. Increased infiltration of these TAMs was positively associated with NET formation and promoted neutrophil NETosis in vitro. Collectively, these findings suggest a potential tumor-promoting crosstalk axis in which P2RY13-negative TAMs facilitate NETosis, highlighting their potential relevance as biomarkers and therapeutic targets across cancers.

## Data Availability

The data presented in this study are available on request from the corresponding author.

## Supplementary Materials

The following supporting information can be downloaded at: https://www.mdpi.com/article/10.3390/cancers18152500/s1, Figure S1: Paired expression analysis of P2RY13 in additional representative cancer types; Figure S2: Pan-cancer Cox regression analyses of the association of P2RY13 with disease-free interval and disease-specific survival; Figure S3: Kaplan–Meier analyses of the association of P2RY13 with DFI and DSS in LUAD, LIHC, and the COADREAD cohort; Figure S4: Pan-cancer correlation analysis between P2RY13 expression and the comprehensive immunomodulatory gene network; Figure S5: Pan-cancer correlation between P2RY13 expression and Immunophenoscore (IPS) metrics; Figure S6: Pan-cancer correlation analysis between P2RY13 expression and tumor-infiltrating cells by using the MCPcounter algorithm; Figure S7: Pan-cancer correlation analysis between P2RY13 expression and tumor microenvironment cell fractions based on the EPIC algorithm; Figure S8: Validation of P2RY13 knockdown and rescue in U937-derived tumor-educated TAM-like macrophages; Figure S9: P2RY13 knockdown in U937-derived tumor-educated TAM-like macrophages promotes neutrophil NET formation; Figure S10: P2RY13 knockdown in U937-derived tumor-educated TAM-like macrophages increases CitH3 release from neutrophils; Figure S11: Positive correlation between P2RY13-negative TAM infiltration and neutrophil infiltration in LUAD, LIHC, and CRC. Table S1: Genes comprising the LUAD-specific P2RY13-negative TAM signature; Table S2: Genes comprising the LIHC-specific P2RY13-negative TAM signature; Table S3: Genes comprising the CRC-specific P2RY13-negative TAM signature. Table S4: Baseline clinicopathological characteristics of the 30 enrolled LUAD patients; Table S5: Baseline clinicopathological characteristics of the 25 enrolled LIHC patients; Table S6: Baseline clinicopathological characteristics of the 20 enrolled CRC patients. Supplementary File S1: Original Image of Western Blot (for Figure 5B and Figure S8).

## Abbreviations

The following abbreviations are used in this manuscript:

NETs	Neutrophil extracellular traps
TIME	Tumor immune microenvironment
TAMs	Tumor-associated macrophages
LUAD	Lung adenocarcinoma
LIHC	Liver hepatocellular carcinoma
CRC	Colorectal cancer
P2RY13	Purinergic receptor P2Y13
MPO	Myeloperoxidase
cit-H3	Citrullinated histone H3
PMA	Phorbol 12-myristate 13-acetate
MHC	Major histocompatibility complex
NK cells	Natural killer cells
