# Supplementary material for: P2RY13-Negative Tumor-Associated Macrophages Promote NETosis and Are Associated with Poor Prognosis Across Multiple Cancers
Source: Cancers (Basel). 2026 Aug 4;18(15):2500. doi: 10.3390/cancers18152500 (PMC13465271; doi:10.3390/cancers18152500)
Supplement: Supplementary file 1 [file cancers-18-02500-s001.zip › cancers-4385386-supplementary .pdf]

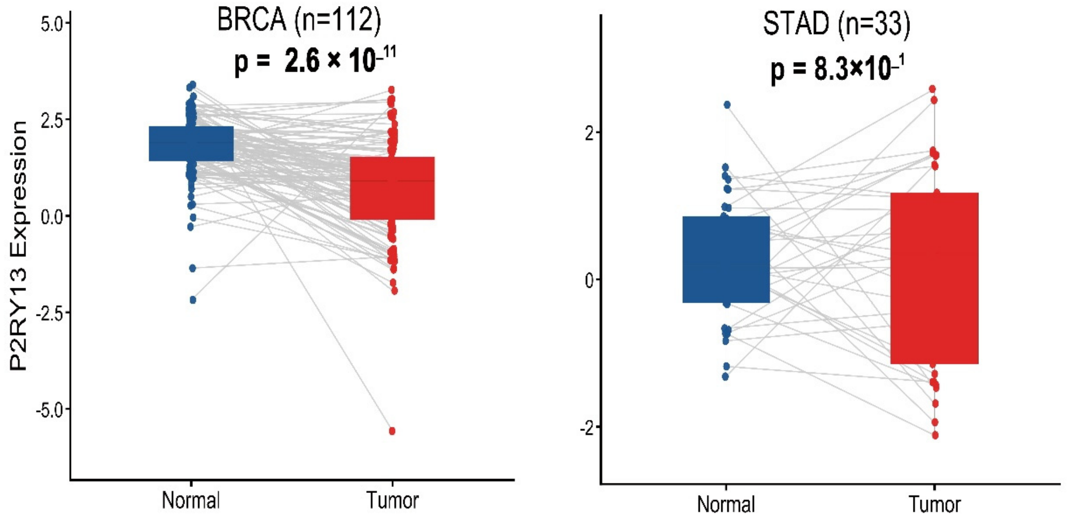

Figure S1: Paired expression analysis of P2RY13 in additional representative cancer types. Paired comparisons of P2RY13 expression between tumor tissues and adjacent normal tissues in breast invasive carcinoma (BRCA) and stomach adenocarcinoma (STAD) are shown. The y-axis represents P2RY13 expression levels, and the x-axis indicates normal and tumor tissues. P2RY13 was significantly downregulated in BRCA tumor tissues compared with paired adjacent normal tissues, whereas no statistically significant difference was observed in STAD. P values are indicated in the figure.

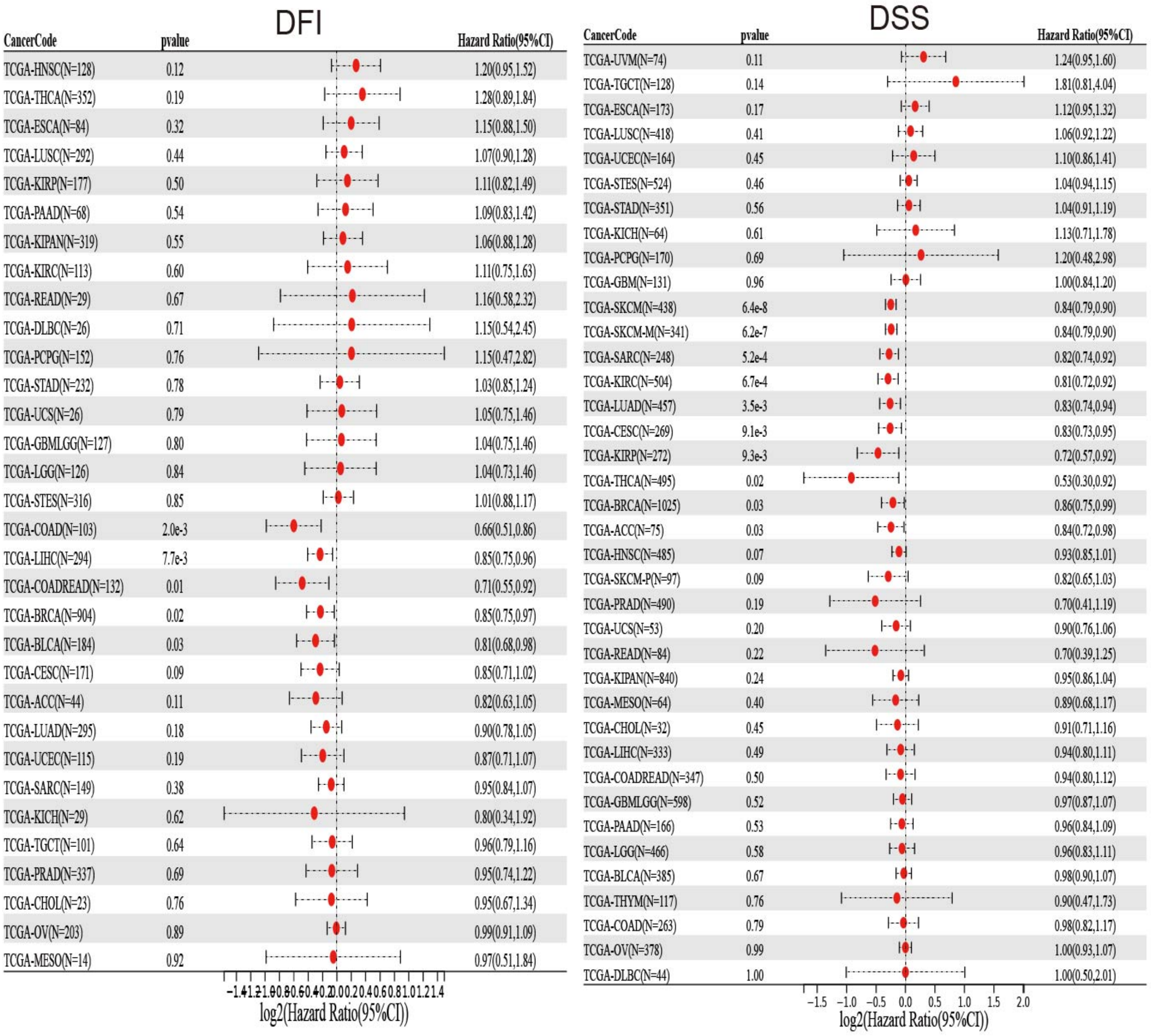

Figure S2: Pan-cancer Cox regression analyses of the association of P2RY13 with disease-free interval and disease-specific survival. Associations between P2RY13 expression and disease-free interval (DFI, *left*), disease-specific survival (DSS, *right*) are shown. The x-axis represents log2-transformed HRs and their 95% CIs, while the y-axis lists different cancer types and corresponding sample sizes. HR < 1 indicates that high P2RY13 expression is associated with a lower risk of recurrence or disease-specific death. P values, HRs, and 95% CIs are indicated in the figure.

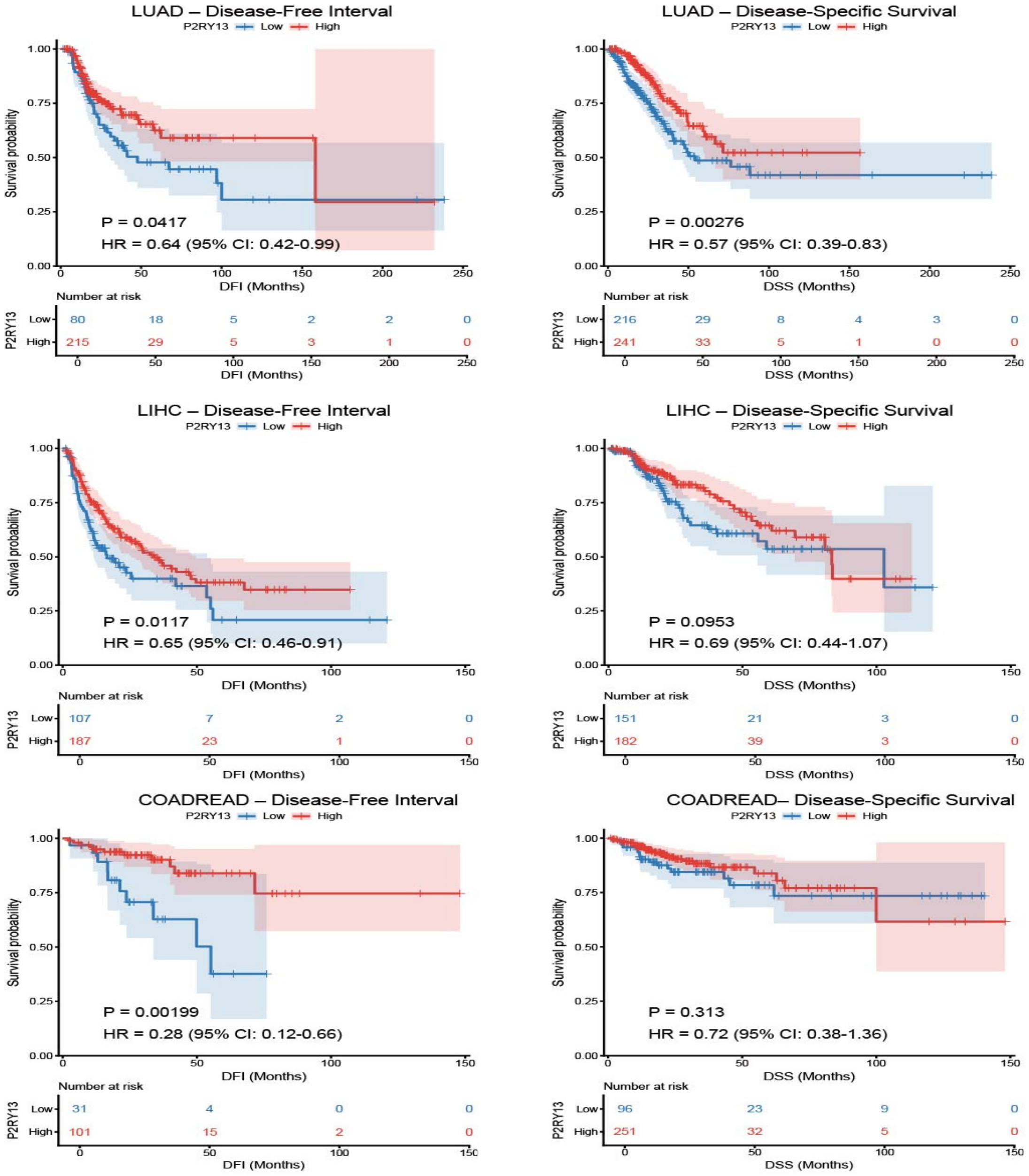

Figure S3: Kaplan-Meier analyses of the association of P2RY13 with DFI and DSS in LUAD, LIHC, and the COADREAD cohort. Patients were divided into high- and low-expression groups based on P2RY13 expression, and the curves display differences in survival probability between the two groups for disease-free interval (DFI) and disease-specific survival (DSS). In LUAD, high P2RY13 expression was associated with improved DFI and DSS; in LIHC and COADREAD, high P2RY13 expression was mainly associated with improved DFI, whereas DSS showed only a non-significant beneficial trend. P values, HRs, and 95% CIs are indicated in the figure.

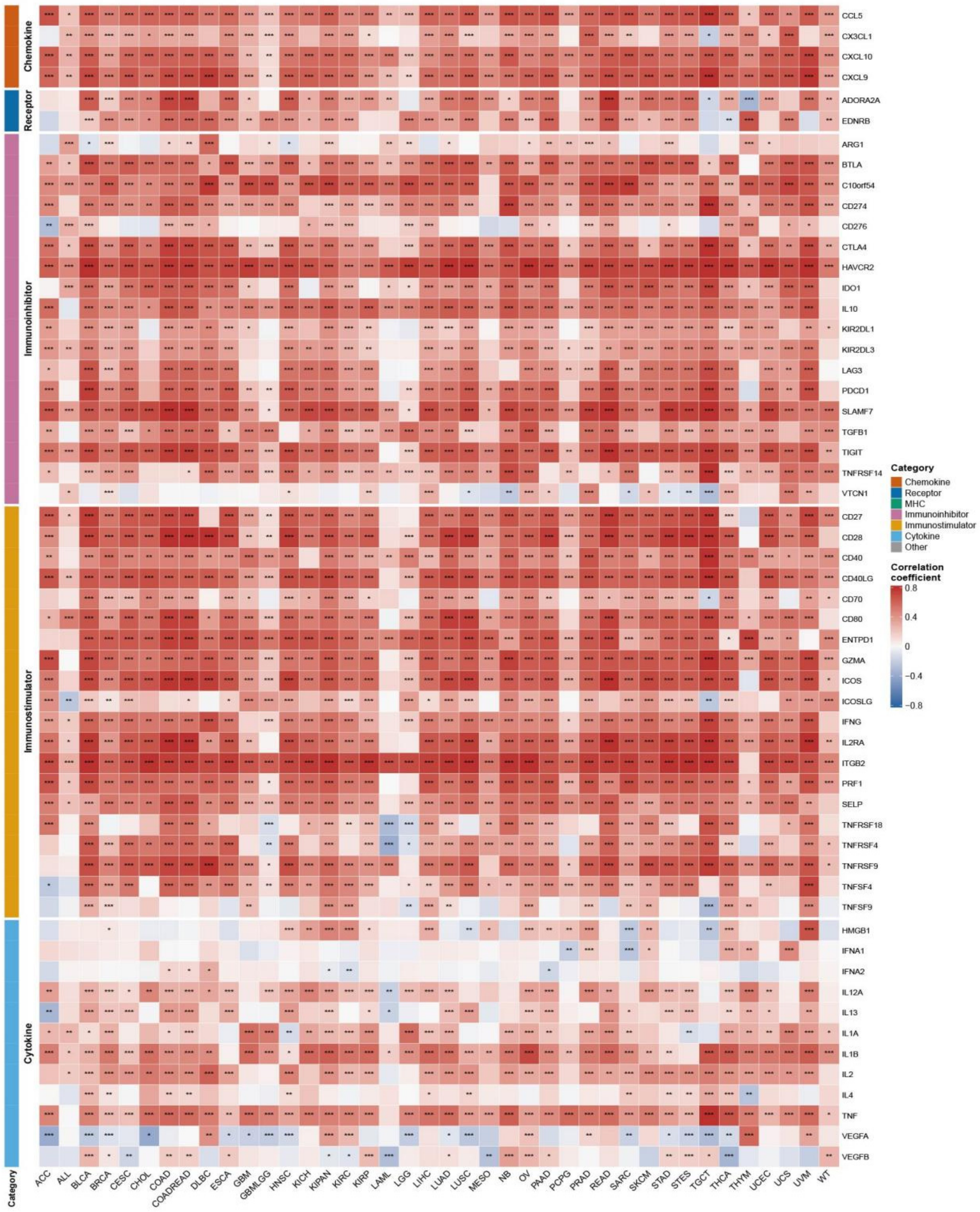

Figure S4: Pan-cancer correlation analysis between P2RY13 expression and the comprehensive immunomodulatory gene network. This heatmap displays Pearson correlation coefficients between P2RY13 expression and various classes of immunomodulatory genes across 33 TCGA cancer types. The Red and blue squares indicate positive and negative correlations, respectively. The results indicate that P2RY13 is closely associated with a broad network of immune activation and compensatory immune checkpoint upregulation in the tumor microenvironment.

* $p < 0.05$; ** $p < 0.01$; *** $p < 0.001$; **** $p < 0.0001$.

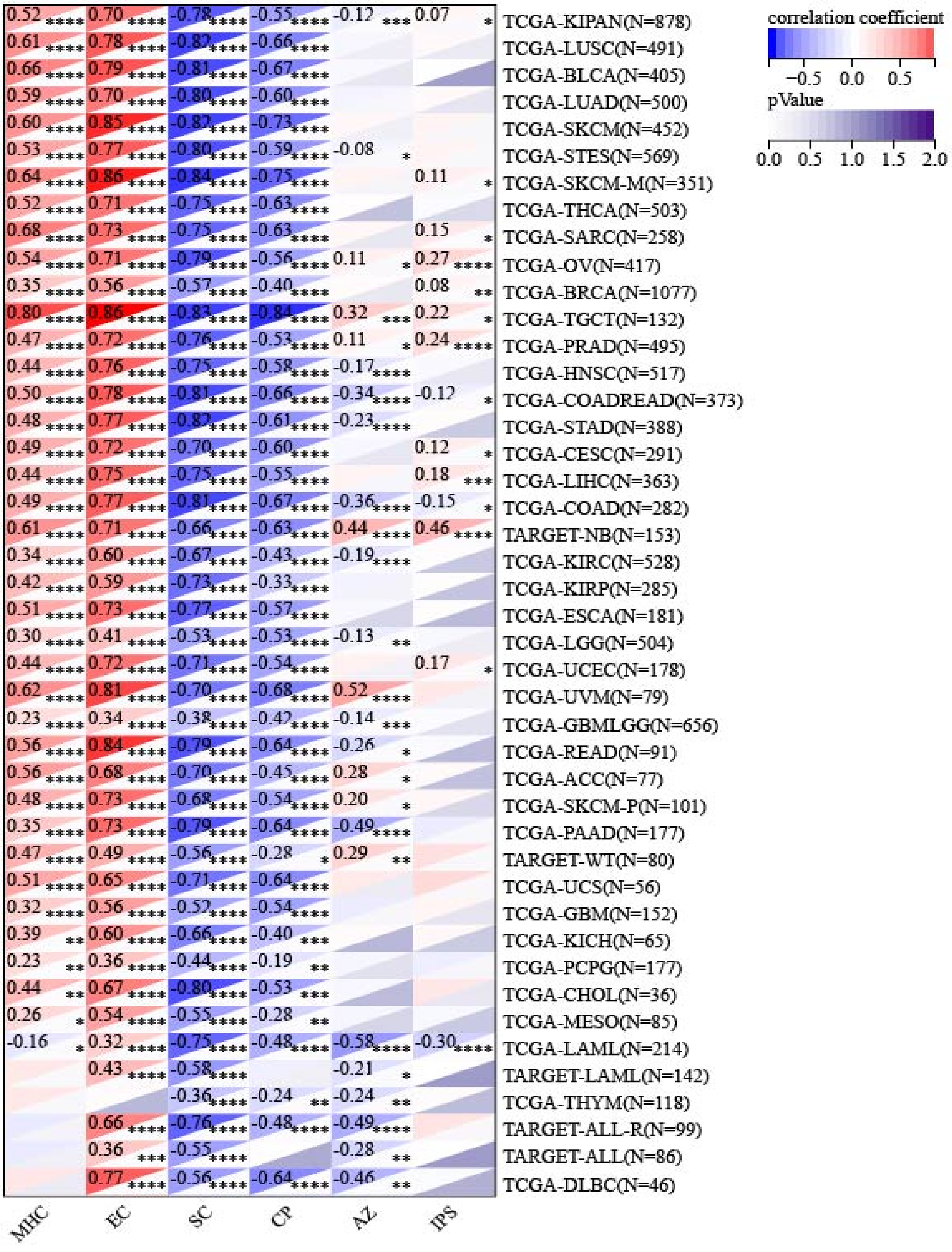

Figure S5. Pan-cancer correlation between P2RY13 expression and Immunophenoscore (IPS) metrics.

P2RY13 expression is generally positively correlated with Major Histocompatibility Complex (MHC) and effector cells (EC, *red*)， but negatively with suppressor cells (SC), immune checkpoints (CP, *blue*) in multiple cancers, suggesting its potential role in mediating antigen presentation and anti-tumor effector cell infiltration.

* $p < 0.05$; ** $p < 0.01$; *** $p < 0.001$; **** $p < 0.0001$.

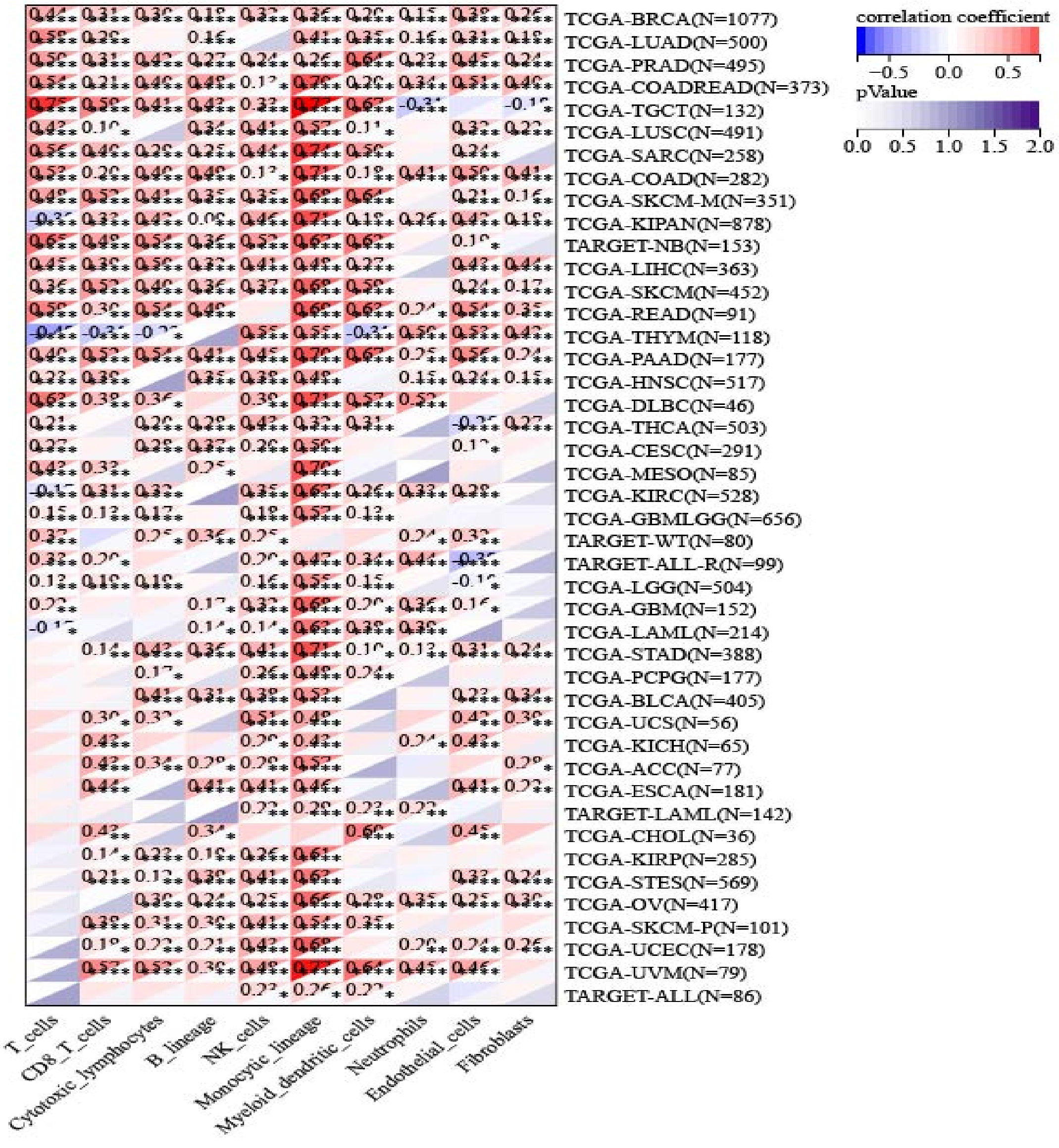

Figure S6: Pan-cancer correlation analysis between P2RY13 expression and tumor-infiltrating cells by using the MCPcounter algorithm. The correlation of P2RY13 expression with the infiltration abundance of various immune and stromal cell subpopulations was estimated by the MCPcounter algorithm. Results suggested positive correlations between P2RY13 and key myeloid and lymphoid subsets, including the monocytic lineage, neutrophils, CD8^{+} T cells, and myeloid dendritic cells.

* $p< 0.05$; ** $p < 0.01$; *** $p < 0.001$; **** $p < 0.0001$.

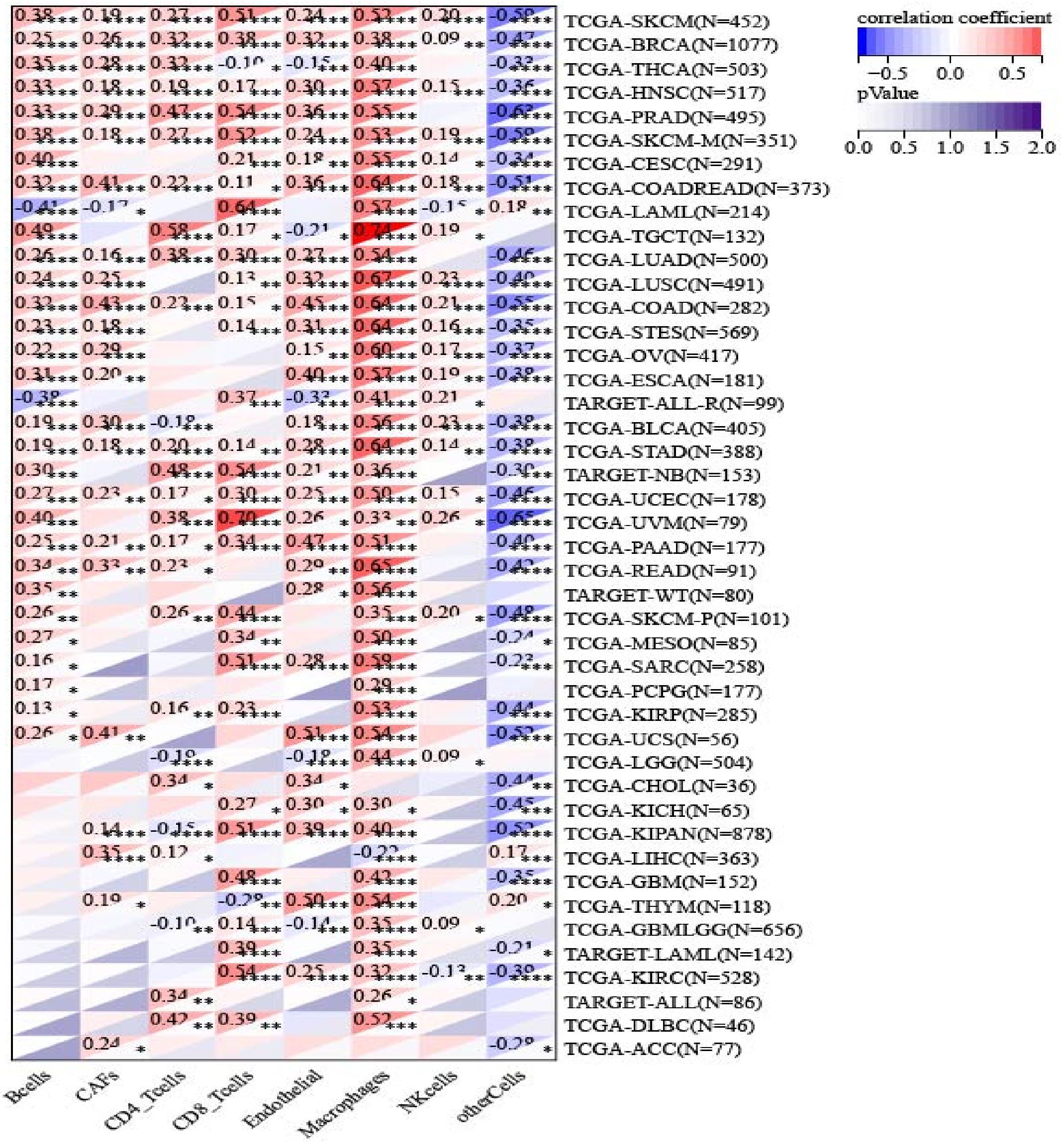

Figure S7: Pan-cancer correlation analysis between P2RY13 expression and tumor microenvironment cell fractions based on the EPIC algorithm. The correlation between P2RY13 expression and the infiltration proportions of macrophages, $CD8^+$ T cells, $CD4^+$ T cells, and cancer-associated fibroblasts (CAFs) was analyzed by using the EPIC algorithm, yielding findings broadly consistent with MCPcounter.

* *p*< 0.05; ** *p* < 0.01; *** *p* < 0.001; **** *p* < 0.0001.

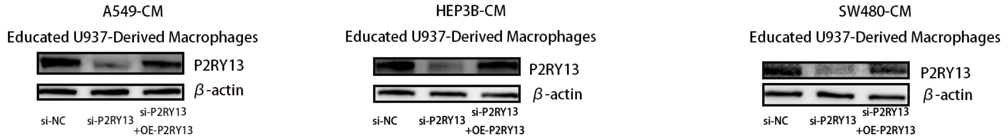

Figure S8. Validation of P2RY13 knockdown and rescue in U937-derived tumor-educated TAM-like macrophages. Western blot analysis of P2RY13 expression in U937-derived TAM-like macrophages educated with TCM from A549, Hep3B, or SW480 cells and transfected with siNC, siP2RY13, or siP2RY13 together with the OE-P2RY13 rescue construct. β-Actin served as the loading control. P2RY13 expression was reduced following siP2RY13 transfection and restored by P2RY13 re-expression. The original image of western blot are shown in Supplementary File S1.

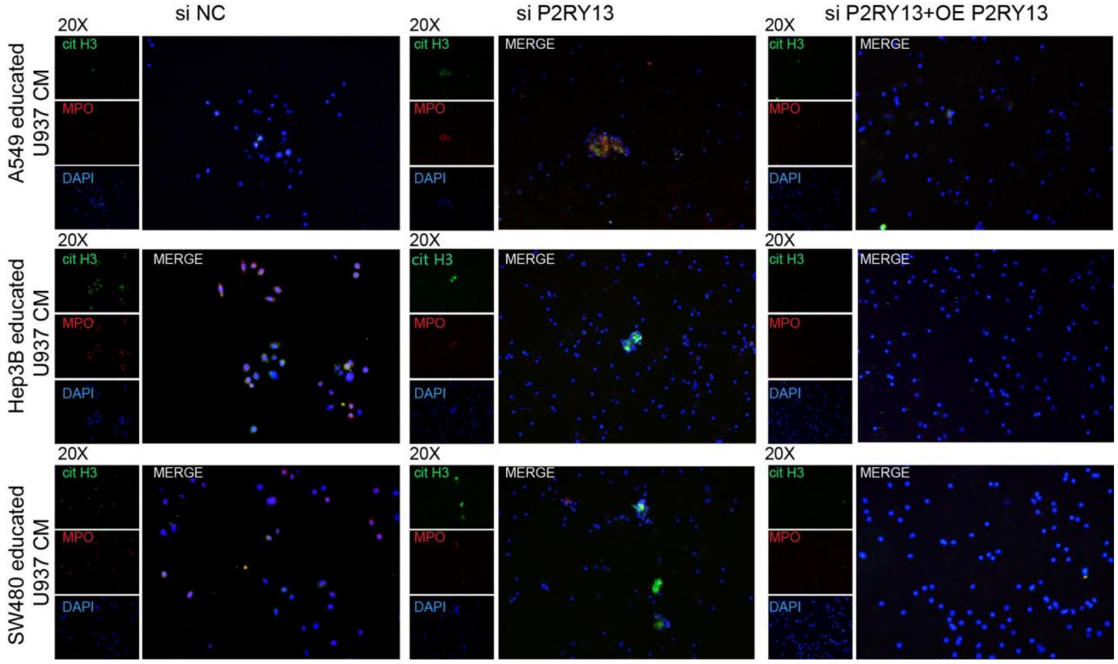

Figure S9. P2RY13 knockdown in U937-derived tumor-educated TAM-like macrophages promotes neutrophil NET formation. Representative immunofluorescence images of neutrophils treated with conditioned medium from the indicated U937-derived TAM-like macrophage groups. NETs were identified by the colocalization of MPO, CitH3, and extracellular DNA signals. Conditioned medium from P2RY13-silenced macrophages increased the formation of MPO^+/$CitH3^+$ extracellular DNA structures across the A549-, Hep3B-, and SW480-educated models, whereas P2RY13 re-expression attenuated this effect.

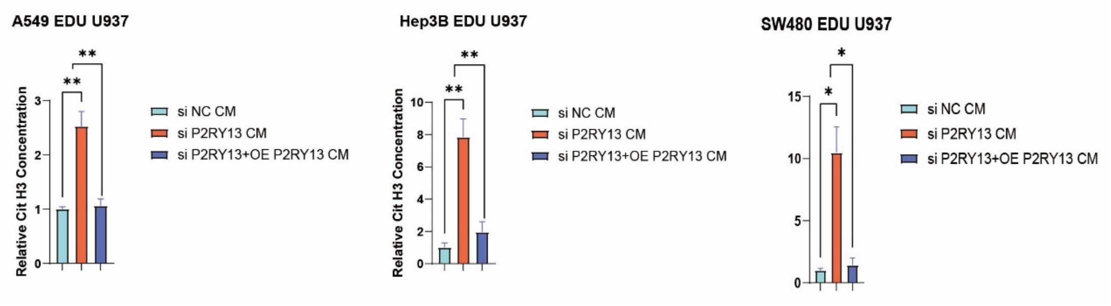

Figure S10. P2RY13 knockdown in U937-derived tumor-educated TAM-like macrophages increases CitH3 release from neutrophils. ELISA quantification of CitH3 levels in the supernatants of neutrophils treated with conditioned medium from the indicated U937-derived TAM-like macrophage groups. CitH3 levels were elevated following treatment with conditioned medium from P2RY13-silenced macrophages and reduced following P2RY13 re-expression across the A549-, Hep3B-, and SW480-educated models. * $p< 0.05$; ** $p < 0.01$; *** $p < 0.001$; **** $p < 0.0001$.

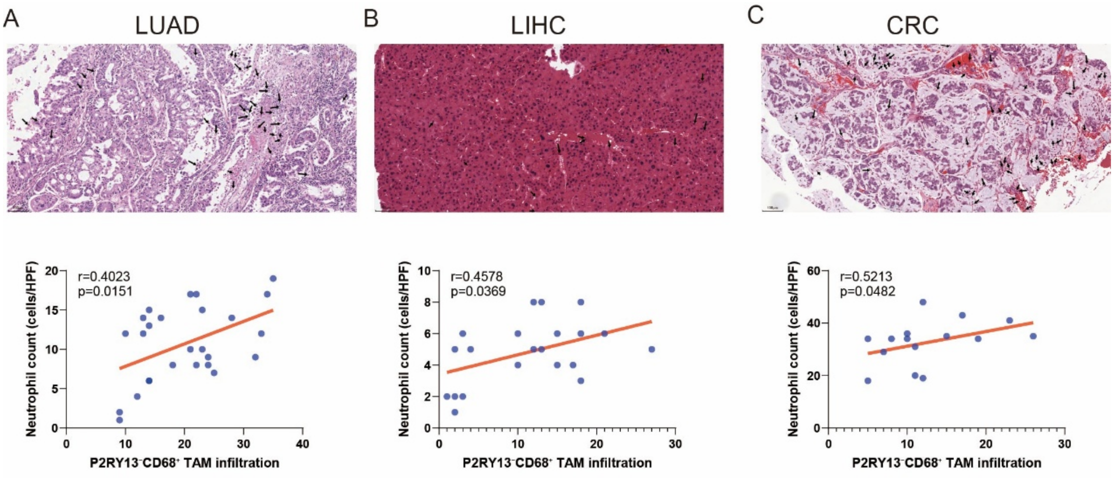

Figure S11. P2RY13-negative TAM infiltration positively correlates with neutrophil infiltration in LUAD, LIHC, and CRC. (A–C) Spearman correlation analyses between P2RY13^{-}CD68^{+} TAM infiltration and histomorphologically quantified neutrophil infiltration in LUAD (A), LIHC (B), and CRC (C) tissue microarrays. Neutrophils were identified in H&E-stained sections by their characteristic segmented or multilobed nuclei and lightly eosinophilic granular cytoplasm. Neutrophil infiltration was expressed as the mean number of neutrophils per high-power field (cells/HPF). Greater infiltration of P2RY13-negative TAMs was significantly associated with increased neutrophil infiltration across all three cancer types. Spearman correlation coefficients (r) and P values are indicated in the corresponding panels.

Table S1. Genes comprising the LUAD-specific P2RY13-negative TAM signature.

Gene symbol	Gene symbol (continued)
SPP1	IER3
BNIP3	CHCHD6
RNASE1	CTSL
S100A12	SEC61G
EREG	S100A9
VCAN	LGALS1
G0S2	SAT1
CCL2	GAPDH
CCL20	CXCL8
TIMP1	FAH
CXCL2	C4orf3
SOD2	

Note: In the scRNA-seq analyses, P2RY13-negative TAMs were operationally defined as TAMs without detectable P2RY13 transcripts.

Table S2. Genes comprising the LIHC-specific P2RY13-negative TAM signature.

Gene symbol	Gene symbol(continued)	Gene symbol(continued)	Gene symbol(continued)
ACTB	IL1RN	RPS12	ZFP36
ACTG1	LAPTM5	RPS15	
ALDOA	LDHA	RPS16	
ALOX5AP	LGALS1	RPS24	
ANXA2	MALAT1	RPS27	
AQP9	MARCO	RPS27L	
ATP5E	MCEMP1	RPS28	
ATP6V1F	MIF	S100A10	
B2M	MT-ATP6	S100A11	
BCL2A1	MT-CO1	S100A4	
C15orf48	MT-CO2	S100A6	
CALM1	MT-CO3	S100A8	
CCL18	MT-CYB	S100A9	
CCL2	MT-ND1	SAT1	
CD44	MT-ND2	SDC2	
CD63	MT-ND3	SDCBP	
CEBPB	MT-ND4	SERF2	
CLEC5A	MT-ND5	SH3BGRL3	
CSTB	MYL6	SLC11A1	
CTSB	NAMPT	SLC16A10	
CTSD	NEAT1	SLC2A3	
CTSL	OAZ1	SOD2	
CXCL2	OLR1	SPP1	
CXCL3	PFDN5	SRGN	
CXCL8	PFN1	TCEB2	
EMP1	PLAUR	THBS1	
EMP3	POMP	TIMP1	
EREG	PTMA	TMSB10	
FCER1G	RGCC	TMSB4X	
FN1	RPL10	TPM4	
FTH1	RPL28	TPT1	
FTL	RPL32	TREM1	
GAPDH	RPL36	TXN	
GCHFR	RPL37A	TYROBP	
GLUL	RPL39	UBA52	
GPX1	RPL41	VCAN	
H3F3A	RPL8	VEGFA	
H3F3B	RPLP1	VIM	
HLA-B	RPLP2	YBX1	

Note: Genes are displayed across four columns for compactness. Together, the columns comprise a single continuous gene list and do not represent separate gene sets. In the scRNA-

seq analyses, P2RY13-negative TAMs were operationally defined as TAMs without detectable P2RY13 transcripts.

Table S3. Genes comprising the CRC-specific P2RY13-negative TAM signature.

Gene symbol	Gene symbol (continued)
AQP9	SOD2
SPP1	TIMP1
SLC16A10	NAMPT
SDC2	CEBPB
CCL2	ALOX5AP
CLEC5A	LDHA
TREM1	TPM4
MCEMP1	CD44
CCL18	CSTB
FN1	GLUL
EMP1	S100A10
IL1RN	S100A6
GCHFR	RPS27L
SLC11A1	TXN
VEGFA	FTH1
VCAN	EMP3
THBS1	SDCBP
KRT19	LGALS1
EREG	CTSD
CXCL3	CTSB
CXCL2	GAPDH
SLC2A3	VIM
C15orf48	S100A4
MARCO	FTL
S100A8	SRGN
BCL2A1	ALDOA
PLAUR	POMP
MIF	
CTSL	
CXCL8	
S100A9	

Note: In the scRNA-seq analyses, P2RY13-negative TAMs were operationally defined as TAMs without detectable P2RY13 transcripts.

Table S4. Baseline clinicopathological characteristics of the 30 enrolled LUAD patients.

Characteristic		Number	Percentage
Gender			
	Female	13	43.30%
	Male	17	56.70%
Age			
	<65 years	15	50.00%
	≥65 years	15	50.00%
Tumor Site			
	Left	9	30.00%
	Right	18	60.00%
	unspecified	3	10.00%
Histology			
	Adenocarcinoma	30	100.00%
Pathological Grade			
	Grade I	2	6.70%
	Grade II	7	23.30%
	Grade III	21	70.00%
Pathological Morphology			
	Central type	7	23.30%
	Peripheral type	3	10.00%
	Unknown	20	66.70%
T stage			
	T1a	0	0.00%
	T1b	6	20.00%
	T1c	8	26.70%
	T2a	5	16.70%
	T2b	5	16.70%
	T3	5	16.70%
	T4	1	3.30%
N stage			
	N0	5	16.70%
	N1	3	10.00%
	N2	3	10.00%
	N3	0	0.00%
	Unknown	19	63.30%

Table S5. Baseline clinicopathological characteristics of the 25 enrolled LIHC patients.

Abbreviation: HCC: Hepatocellular carcinoma, ICCA: Intrahepatic cholangiocarcinoma, Sus. ICCA:

**Characteristic**		**Number**	**Percentage**
Gender			
	Female	5	20.00%
	Male	20	80.00%
Age			
	<60 years	16	64.00%
	≥60 years	8	32.00%
	Unknown	1	4.00%
Tumor Site			
	Left	6	24.00%
	Right	14	56.00%
	unspecified	4	16.00%
	Unknown	1	4.00%
Histology			
	HCC	23	92.00%
	ICCA	1	4.00%
	Sus. ICCA	1	4.00%
Pathological Grade			
	Grade I	1	4.00%
	Grade II	15	60.00%
	Grade III	7	28.00%
	Unknown	2	8.00%
Tumor Size			
	≤5 cm	12	48.00%
	>5 cm	11	44.00%
	Unknown	2	8.00%

Suspected intrahepatic cholangiocarcinoma

Table S6. Baseline clinicopathological characteristics of the 20 enrolled CRC patients.

Characteristic		Number	Percentage
Gender			
	Female	9	45.00%
	Male	11	55.00%
Age			
	<60 years	14	70.00%
	≥60 years	5	25.00%
	Unknown	1	5.00%
Tumor Site			
	Sigmoid colon	6	30.00%
	Ascending colon	4	20.00%
	Transverse colon	3	15.00%
	Right hemicolon	2	10.00%
	Colon, unspecified	2	10.00%
	Splenic flexure of transverse colon	1	5.00%
	Left hemicolon	1	5.00%
	Ascending colon and ileocecal region	1	5.00%
	Descending-sigmoid junction	1	5.00%
	Transverse colon / other recorded site	1	5.00%
Histology			
	Mucinous adenocarcinoma	20	100.00%
Pathological Grade			
	Grade I	0	0.00%
	Grade II	8	40.00%
	Grade III	12	60.00%
N stage			
	N0	5	25.00%
	N1a	2	10.00%
	N1b	3	15.00%
	N2a	4	20.00%
	N2b	6	30.00%
M stage			
	M0	20	100.00%
