# Supplementary material for: P2RY13-Negative Tumor-Associated Macrophages Promote NETosis and Are Associated with Poor Prognosis Across Multiple Cancers
Source: Cancers (Basel). 2026 Aug 4;18(15):2500. doi: 10.3390/cancers18152500 (PMC13465271; doi:10.3390/cancers18152500)
Supplement: Supplementary file 1 [file cancers-18-02500-s001.zip › cancers-4385386-Supplementary File S1.pdf]

Figure 5B

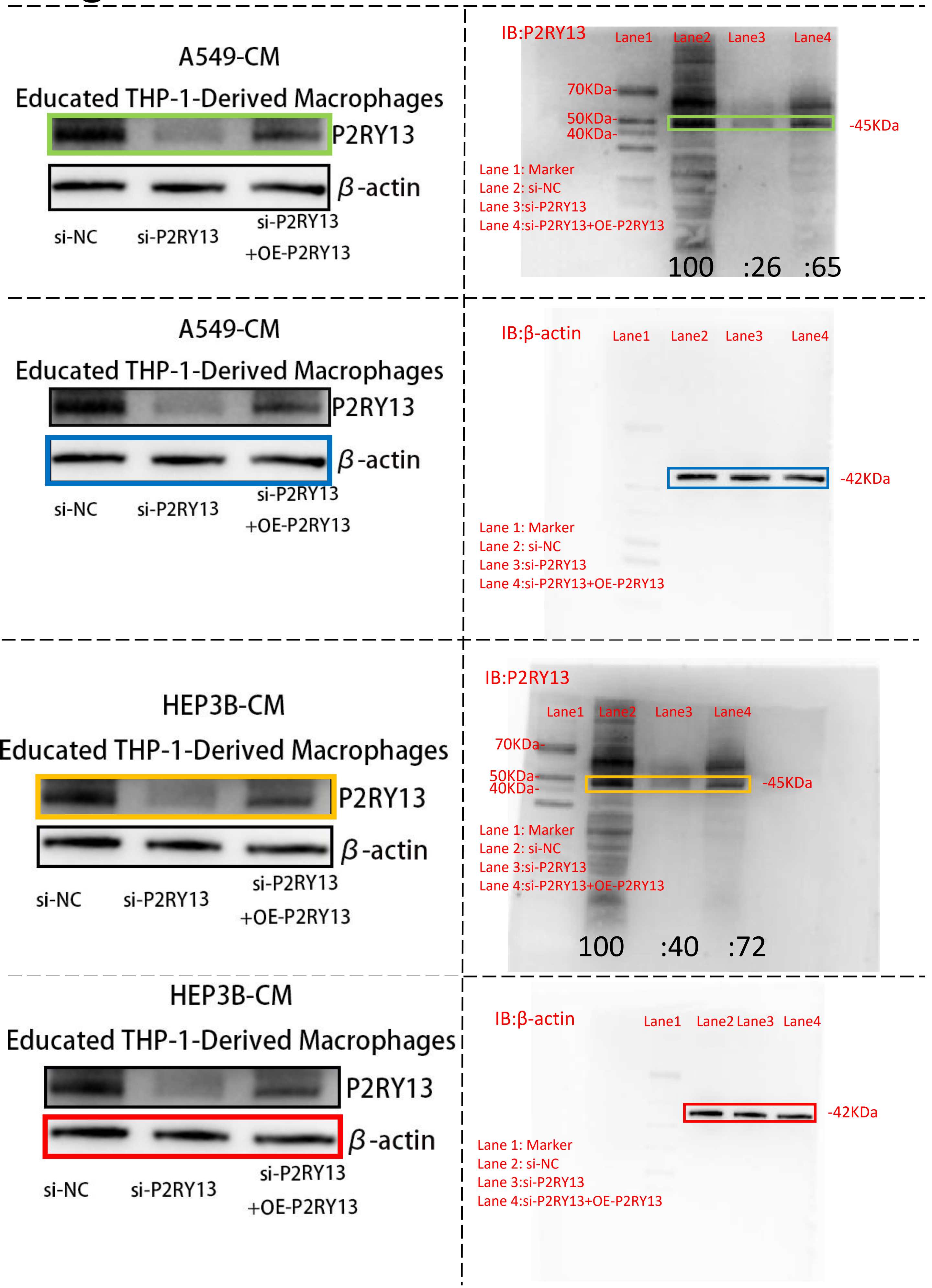
A549-CM
Educated THP-1-Derived Macrophages
P2RY13
β-actin
si-NC
si-P2RY13
si-P2RY13
+OE-P2RY13
IB:P2RY13
Lane1
Lane2
Lane3
Lane4
70KDa-
50KDa-
40KDa-
-45KDa
Lane 1: Marker
Lane 2: si-NC
Lane 3:si-P2RY13
Lane 4:si-P2RY13+OE-P2RY13
100 :26 :65
A549-CM
Educated THP-1-Derived Macrophages
P2RY13
β-actin
si-NC
si-P2RY13
si-P2RY13
+OE-P2RY13
IB:β-actin
Lane1
Lane2
Lane3
Lane4
-42KDa
Lane 1: Marker
Lane 2: si-NC
Lane 3:si-P2RY13
Lane 4:si-P2RY13+OE-P2RY13
HEP3B-CM
Educated THP-1-Derived Macrophages
P2RY13
β-actin
si-NC
si-P2RY13
si-P2RY13
+OE-P2RY13
IB:P2RY13
Lane1
Lane2
Lane3
Lane4
70KDa-
50KDa-
40KDa-
-45KDa
Lane 1: Marker
Lane 2: si-NC
Lane 3:si-P2RY13
Lane 4:si-P2RY13+OE-P2RY13
100 :40 :72
HEP3B-CM
Educated THP-1-Derived Macrophages
P2RY13
β-actin
si-NC
si-P2RY13
si-P2RY13
+OE-P2RY13
IB:β-actin
Lane1
Lane2
Lane3
Lane4
-42KDa
Lane 1: Marker
Lane 2: si-NC
Lane 3:si-P2RY13
Lane 4:si-P2RY13+OE-P2RY13

Figure 5B

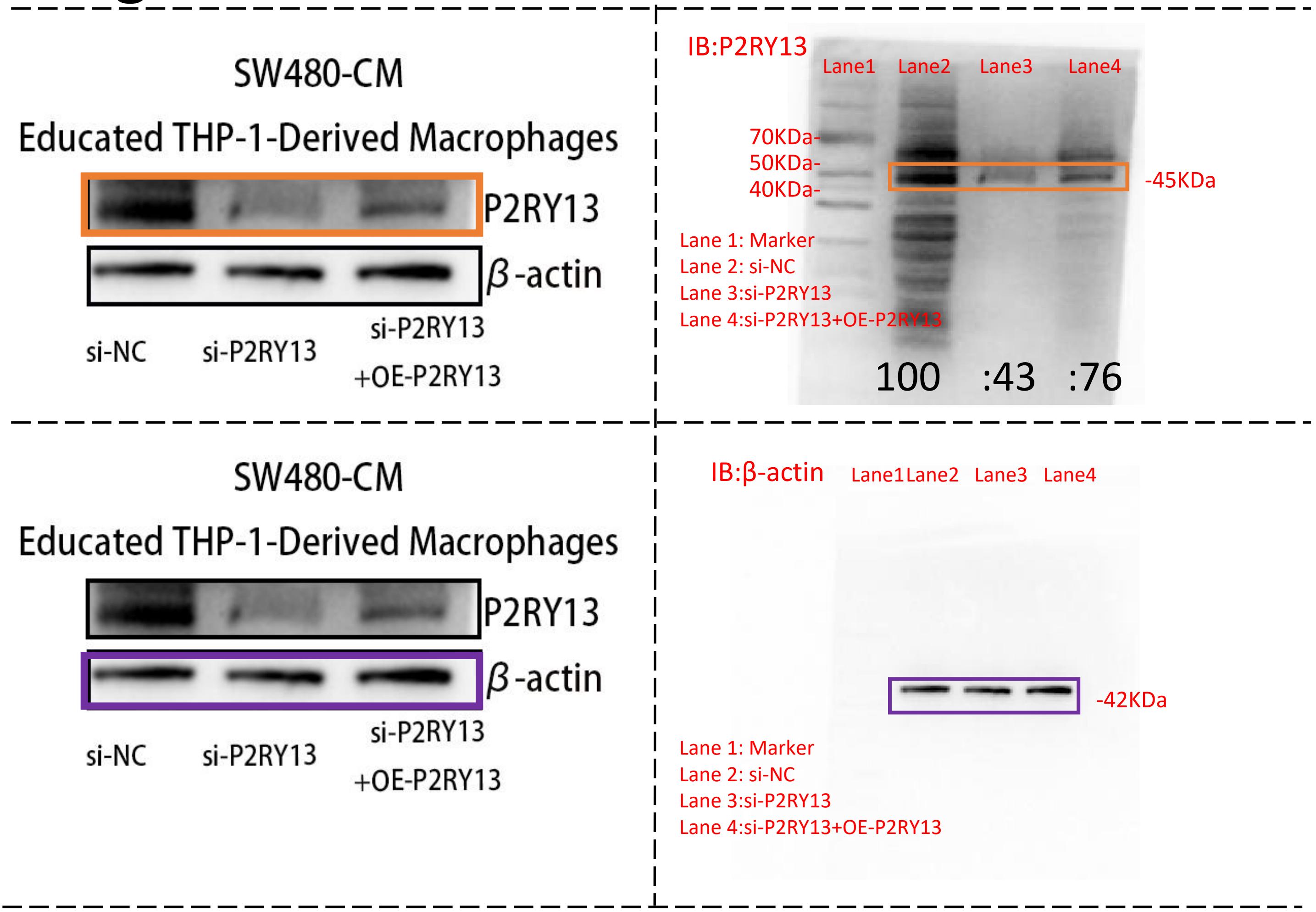
SW480-CM
Educated THP-1-Derived Macrophages
P2RY13
β-actin
si-NC
si-P2RY13
si-P2RY13
+OE-P2RY13
IB:P2RY13
Lane1
Lane2
Lane3
Lane4
70KDa-
50KDa-
40KDa-
-45KDa
Lane 1: Marker
Lane 2: si-NC
Lane 3:si-P2RY13
Lane 4:si-P2RY13+OE-P2RY13
100
:43
:76
SW480-CM
Educated THP-1-Derived Macrophages
P2RY13
β-actin
si-NC
si-P2RY13
si-P2RY13
+OE-P2RY13
IB:β-actin
Lane1
Lane2
Lane3
Lane4
-42KDa
Lane 1: Marker
Lane 2: si-NC
Lane 3:si-P2RY13
Lane 4:si-P2RY13+OE-P2RY13

Figure S8

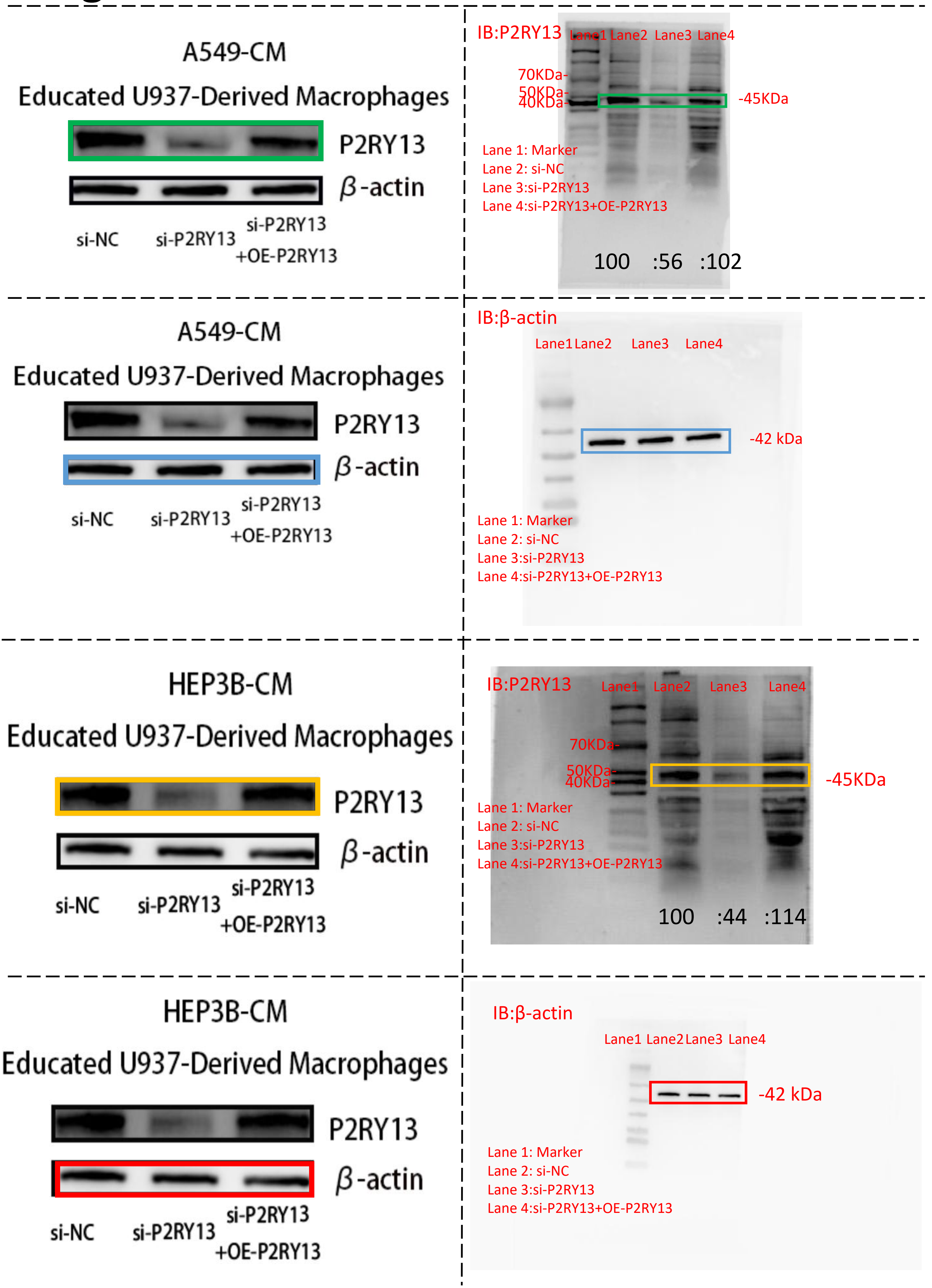
A549-CM
Educated U937-Derived Macrophages
P2RY13
β-actin
si-NC
si-P2RY13
si-P2RY13
+OE-P2RY13
IB:P2RY13
Lane1 Lane2 Lane3 Lane4
70KDa-
50KDa-
40KDa-
-45KDa
Lane 1: Marker
Lane 2: si-NC
Lane 3:si-P2RY13
Lane 4:si-P2RY13+OE-P2RY13
100 :56 :102
A549-CM
Educated U937-Derived Macrophages
P2RY13
β-actin
si-NC
si-P2RY13
si-P2RY13
+OE-P2RY13
IB:β-actin
Lane1 Lane2 Lane3 Lane4
-42 kDa
Lane 1: Marker
Lane 2: si-NC
Lane 3:si-P2RY13
Lane 4:si-P2RY13+OE-P2RY13
HEP3B-CM
Educated U937-Derived Macrophages
P2RY13
β-actin
si-NC
si-P2RY13
si-P2RY13
+OE-P2RY13
IB:P2RY13
Lane1 Lane2 Lane3 Lane4
70KDa-
50KDa-
40KDa-
-45KDa
Lane 1: Marker
Lane 2: si-NC
Lane 3:si-P2RY13
Lane 4:si-P2RY13+OE-P2RY13
100 :44 :114
HEP3B-CM
Educated U937-Derived Macrophages
P2RY13
β-actin
si-NC
si-P2RY13
si-P2RY13
+OE-P2RY13
IB:β-actin
Lane1 Lane2 Lane3 Lane4
-42 kDa
Lane 1: Marker
Lane 2: si-NC
Lane 3:si-P2RY13
Lane 4:si-P2RY13+OE-P2RY13

Figure S8

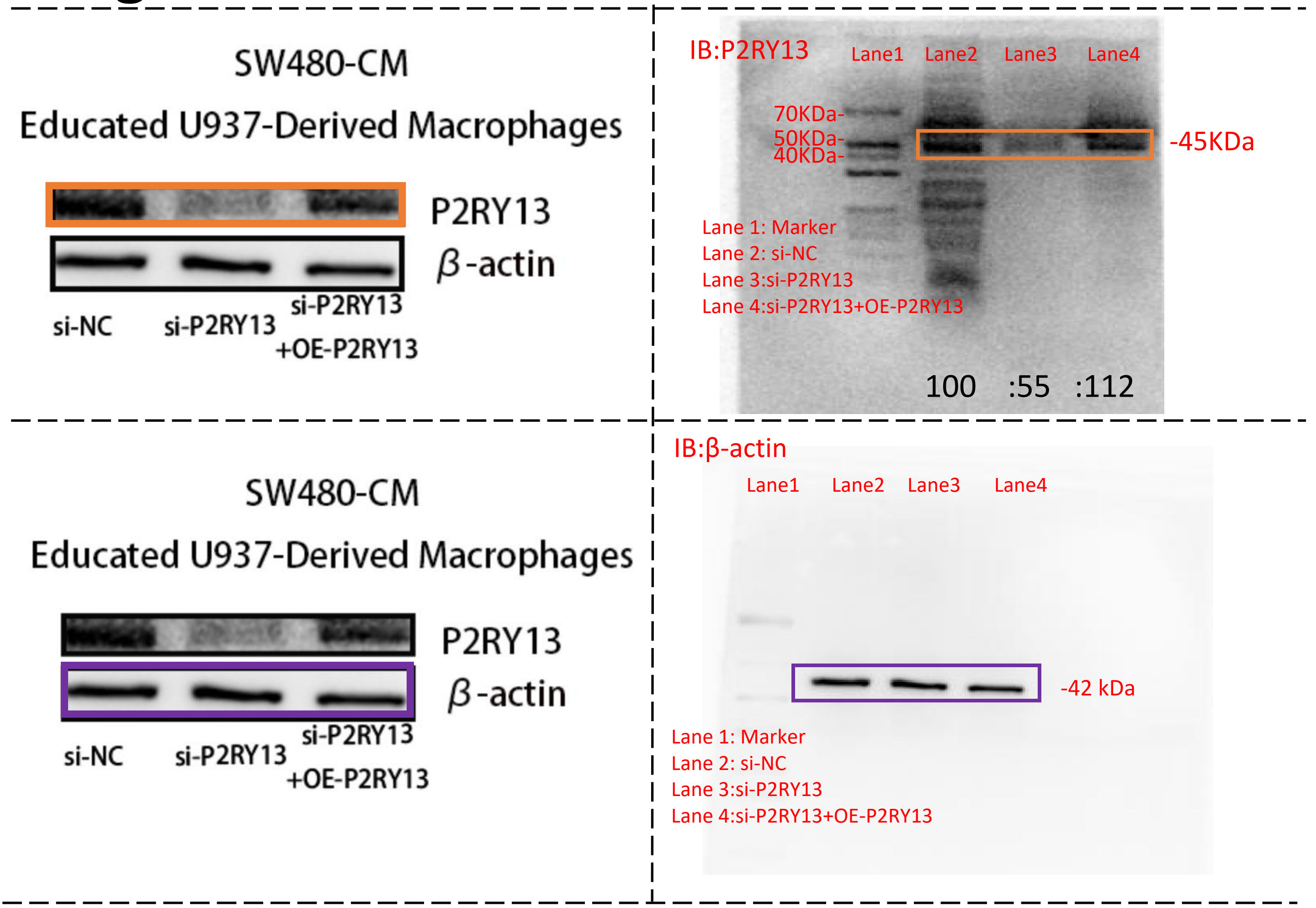

SW480-CM
Educated U937-Derived Macrophages
P2RY13
β-actin
si-NC
si-P2RY13
si-P2RY13
+OE-P2RY13
IB:P2RY13
Lane1
Lane2
Lane3
Lane4
70KDa-
50KDa-
40KDa-
-45KDa
Lane 1: Marker
Lane 2: si-NC
Lane 3:si-P2RY13
Lane 4:si-P2RY13+OE-P2RY13
100 :55 :112
SW480-CM
Educated U937-Derived Macrophages
P2RY13
β-actin
si-NC
si-P2RY13
si-P2RY13
+OE-P2RY13
IB:β-actin
Lane1
Lane2
Lane3
Lane4
-42 kDa
Lane 1: Marker
Lane 2: si-NC
Lane 3:si-P2RY13
Lane 4:si-P2RY13+OE-P2RY13
